# Physiological and biochemical characteristics and microbial responses of *Medicago sativa* (Fabales: Fabaceae) varieties with different resistance to atrazine stress

**DOI:** 10.3389/fmicb.2024.1447348

**Published:** 2024-08-16

**Authors:** Yingao Li, Jiading Lu, Chunyang Dong, Haojie Wang, Boshuai Liu, Defeng Li, Yalei Cui, Zhichang Wang, Sen Ma, Yinghua Shi, Chengzhang Wang, Xiaoyan Zhu, Hao Sun

**Affiliations:** Henan Key Laboratory of Innovation and Utilization of Grassland Resources, College of Animal Science and Technology, Henan Agricultural University, Zhengzhou, China

**Keywords:** alfalfa, atrazine, abiotic stress, oxidative damage, inter-root microorganisms

## Abstract

Atrazine, a commonly employed herbicide for corn production, can leave residues in soil, resulting in photosynthetic toxicity and impeding growth in subsequent alfalfa (*Medicago sativa* L.) crops within alfalfa-corn rotation systems. The molecular regulatory mechanisms by which atrazine affects alfalfa growth and development, particularly its impact on the microbial communities of the alfalfa rhizosphere, are not well understood. This study carried out field experiments to explore the influence of atrazine stress on the biomass, chlorophyll content, antioxidant system, and rhizosphere microbial communities of the atrazine-sensitive alfalfa variety WL-363 and the atrazine-resistant variety JN5010. The results revealed that atrazine significantly reduced WL-363 growth, decreasing plant height by 8.58 cm and root length by 5.42 cm (*p* < 0.05). Conversely, JN5010 showed minimal reductions, with decreases of 1.96 cm in height and 1.26 cm in root length. Chlorophyll content in WL-363 decreased by 35% under atrazine stress, while in JN5010, it was reduced by only 10%. Reactive oxygen species (ROS) accumulation increased by 60% in WL-363, compared to a 20% increase in JN5010 (*p* < 0.05 for both). Antioxidant enzyme activities, such as superoxide dismutase (SOD) and catalase (CAT), were significantly elevated in JN5010 (*p* < 0.05), suggesting a more robust defense mechanism. Although the predominant bacterial and fungal abundances in rhizosphere soils remained generally unchanged under atrazine stress, specific microbial groups exhibited variable responses. Notably, *Promicromonospora* abundance declined in WL-363 but increased in JN5010. FAPROTAX functional predictions indicated shifts in the abundance of microorganisms associated with pesticide degradation, resistance, and microbial structure reconstruction under atrazine stress, displaying different patterns between the two varieties. This study provides insights into how atrazine residues affect alfalfa rhizosphere microorganisms and identifies differential microbial responses to atrazine stress, offering valuable reference data for screening and identifying atrazine-degrading bacteria.

## Introduction

Alfalfa (*Medicago sativa* L.), often termed the “king of forages,” is pivotal in agriculture and livestock production due to its high yield, superior quality, wide adaptability, and resistance to biotic and abiotic stress (Adhikari and Missaoui, 2017; Gu et al., 2018; Song et al., 2022). Its robust root system can access deep soil to obtain water sources, improve soil fertility through nitrogen fixation, and aid in ecological enhancement and erosion control. However, challenges such as substantial water requirements, susceptibility to autotoxicity, and the need for periodic crop rotation highlight the sustainability issues associated with alfalfa cultivation (Chon et al., 2002; Peng et al., 2022; Wang C. et al., 2022; Wang M. et al., 2022). Studies suggest that intercropping alfalfa with crops like maize can optimize land use, improve crop quality and yield, and increase economic benefits (Temel et al., 2017; Peng et al., 2024).

Atrazine, a prevalent herbicide in maize cultivation, disrupts photosynthesis by impeding electron transport and interfering with photosystem II, thereby reducing CO_2_ fixation efficiency and inhibiting weed growth (Zhang et al., 2017; Fan et al., 2018). Extensive research has demonstrated atrazine’s profound effects on plant physiology and biochemistry, including alterations in growth parameters, chlorophyll content, enzymatic activities, and microbial diversity in rhizosphere (Fox et al., 2007). Notably, atrazine significantly reduces the relative growth rate of emergent plants, exposing them to oxidative stress and potential cellular damage (Wang et al., 2015). Furthermore, it impedes soybean growth by impairing glutamate synthase activity and decreasing total nitrogen (TN) content within the plant (Jiang et al., 2024). Atrazine-induced modifications in root nodule structure, leghemoglobin content, and rhizobia diversity in the rhizosphere have also been noted. In pearl millet (*Pennisetum glaucum* L.), increased atrazine levels have been linked with elevated production of reactive oxygen species (ROS) and malondialdehyde (MDA) in leaves, indicative of oxidative damage (Erinle et al., 2016). Moreover, atrazine affects enzymatic activity by enhancing ascorbate peroxidase (APX) and peroxidase (POD) activities, while suppressing those of catalase (CAD) and superoxide dismutase (SOD). These enzymatic alterations result in reduced chlorophyll content, an increased chlorophyll (a/b) ratio, and diminished biomass, plant height, and chlorophyll fluorescence parameters (Sher et al., 2021). Additionally, atrazine, as a component of herbicide mixtures, temporarily inhibits soil microbial community activities including dehydrogenase, urease, and sucrase, essential for the decomposition of soil organic matter and nutrient cycling (Liu et al., 2020). It also alters microbial community structures, notably increasing fungal community abundance and significantly impacting key functional microbial communities such as *Pseudomonas* and *Enterobacteriaceae* (Ma et al., 2023).

Atrazine is continuously used in farmland due to its broad-spectrum, high efficiency, economy, and selectivity advantages. However, the long-term residual effect of herbicide on corn and other crops often causes phytotoxicity to sensitive crops such as alfalfa due to the residual soil after herbicide application, which inhibits the normal growth of alfalfa (Moore and Kröger, 2010; Ibrahim et al., 2013; Zhang et al., 2017). Consequently, atrazine toxicity poses a significant concern in the maize-alfalfa cropping pattern (Zhang et al., 2016; Erinle et al., 2018). Recent years have seen comprehensive investigations into the mechanisms of atrazine degradation and detoxification in alfalfa. Advanced techniques, such as RNA sequencing, have demonstrated that alfalfa can metabolize and degrade atrazine via various pathways, including redox, binding, and hydrolysis reactions. Notably, certain genes such as glutathione *S*-transferases (GSTs) and ABC transporter proteins exhibit significant up-regulation during atrazine detoxification (Zhang et al., 2016, 2017; Yang et al., 2022). However, systematic comparative studies on different resistant varieties are scarce, and the dynamic shifts in inter-root microbial communities and their association with plant resistance have not received adequate attention. This study investigates key inter-root microorganisms involved in atrazine degradation by comparing the growth, antioxidant capacity, and dynamics of inter-root microbial communities among different resistant alfalfa varieties (the resistant variety, JN 5010, and the sensitive variety, WL-363), aiming to elucidate their functional roles in enhancing alfalfa’s resistance to atrazine stress.

In our preceding study, we differentiated atrazine-sensitive from atrazine-tolerant alfalfa varieties through comparative field experiments. The present study aims to elucidate the inter-root variations in response to atrazine stress by examining growth indices, antioxidant enzyme activities, and physiological parameters across alfalfa varieties with differing levels of atrazine tolerance. Furthermore, 16S rRNA sequencing was conducted to analyze the rhizosphere microbiome. Our objectives include identifying variation patterns in the microbiota associated with atrazine stress response, uncovering the regulatory mechanisms behind atrazine tolerance in diverse alfalfa varieties, and providing reference data for the screening and identification of atrazine-degrading bacteria (Supplementary Figure S1).

## Materials and methods

### Experimental design

The experimental field was located at the Henan Agricultural University, Science and Education Experimental Park campus (113°56′ E, 35°6′ N), China. The average temperature, annual precipitation, and atmospheric pressure were 15.4°C, 1245 mm, and 120.8 kPa, respectively. Moreover, the annual sunshine duration and frost-free period were approximately 2,000 h and 224 days, respectively. Prior to the assay, the chemical properties of the surface soil (0–20 cm) included 14.20 g·kg^−1^ of soil organic carbon (SOC), 71.60 mg·kg^−1^ of alkaline hydrolyzable nitrogen (AN), 17.70 mg·kg^−1^ of available phosphorus (AP), and 113.00 mg·kg^−1^ of available potassium (AK). The predominant weeds in the maize field were *Eleusine indica* (L.) Gaertn. and *Digitaria sanguinalis* (L.) Scop. Based on high performance liquid chromatography-mass spectrometry (HPLC-MS), the residual level of atrazine in soil was determined at the end of September when corn was harvested, with a residual amount of 0.76 μg·kg^−1^. The atrazine-tolerant alfalfa variety “JINNENG 5010” (also known as JN5010) and the atrazine-sensitive alfalfa variety “WL-363” were sown on October 11 in plots measuring 15 m^2^ (7.5 m × 2 m), with a row spacing of 0.2 m, a seeding rate of 22.5 kg·ha^−1^, and a sowing depth of 2 cm. Throughout the experiment, the alfalfa was managed with standard fertilization and irrigation practices. The experimental design employed a randomized block method with four replications for each treatment.

### Plant sampling and analysis

During the spring regrowth period, 20 alfalfa plants were randomly selected from each treatment group for the collection of root, stem, and leaf tissues. The samples were immediately placed into pre-cooled freezing tubes, sealed in liquid nitrogen, and stored in a −80°C freezer. These samples were subsequently used for enzyme activity and physiological index assays. In both the atrazine-treated and control plots, 20 uniformly grown alfalfa plants were randomly selected. After soil removal, the length of the above-ground part and the natural elongation of the main root were measured. The weight of each plant was recorded to determine the root-to-shoot ratio. Chlorophyll content was measured using the ethanol immersion method (Arnon, 1949; Porra et al., 1989). A 0.1 g sample of fresh leaves was macerated in 5 mL of 80% ethanol for 24 h in darkness. Following centrifugation at 5000 g for 10 min, the supernatant was collected. The absorbance was measured at 649 nm and 665 nm using a UV–Vis spectrophotometer to calculate the content of chlorophyll a, chlorophyll b, and total chlorophyll. The formulas used for these calculations were as follows (Arnon, 1949; Porra et al., 1989; Wellburn, 1994):

$$\mathrm{Chlorophyll}\;a\;\left(\mathrm{mg}/\mathrm{mL}\right)=13.95A_{665}-6.88A_{649}$$

$$\mathrm{Chlorophyll}\;b\;\left(\mathrm{mg}/\mathrm{mL}\right)=24.96A_{649}-7.32A_{665}$$

$$\mathrm{Total}\;\mathrm{chlorophyll}\;\left(\mathrm{mg}/\mathrm{mL}\right)=18.08A_{649}+6.63A_{665}$$

### Enzyme activity determination and physiological indicator detection

According to the kit manuals (BC0090, BC0170, BC0355, BC0200, BC0020; Beijing Solaibao Technology Co., Ltd., China), the activities of peroxidase (POD), superoxide dismutase (SOD), Glutathione *S*-transferase (GST), catalase (CAT), and malondialdehyde (MDA) were determined. Subsequently, oxidative damage indicators, H_2_O_2_ and O_2_^−^, were detected in the sampled tissues according to the respective kit manuals (BC3595, BC1290; Beijing Solaibao Technology Co., Ltd., China). Lastly, the total antioxidant capacity (T-AOC) was measured following the protocol in the kit manual (BC1310; Beijing Solaibao Technology Co., Ltd., China). Each indicator was tested in triplicate to ensure accuracy and repeatability.

### Rhizosphere soil sampling and analysis

#### Rhizosphere soil collection

During the spring regrowth period, soil samples from the inter-root zone (within 0.5 cm of the root system) were collected from two alfalfa varieties using sterile brushes. Five sampling points were established in each plot, and the collected soil samples were homogeneously mixed. These mixed samples were then flash-frozen in liquid nitrogen and stored at −80°C in a refrigerator for subsequent microbiota analysis. For each variety, three replicate plots were established under each treatment.

#### DNA extraction and PCR amplification

Soil samples for DNA extraction were collected during the regreening stage. Microbial DNA was extracted from 0.5 g of each soil sample using the Omega Mag-Bind Soil DNA Kit (Omega Inc., United States). The quality of the extracted genomic DNA was assessed by 1% agarose gel electrophoresis, and its concentration and purity were measured with a NanoDrop2000 spectrophotometer. PCR amplification utilized *TransStart*^®^ *FastPfu* DNA Polymerase (TransGen AP221-02) and an ABI GeneAmp^®^ 9700 PCR system. For the bacterial analysis, the 16S rRNA gene V3-V4 variable region was amplified with primer pair 338F (5′-ACTCCTACGGGGAGGCAGCAG-3′) and 806R (5′-GGACTACHVGGGGTWTCTAAT-3′). The fungal locus was targeted with primers ITS1 (5′-CTTGGTCATTTAGAGGAAGTAA-3′) and ITS2 (5′-TGCGTTCTTCATCGATGC-3′). The 20 μL PCR reaction mixture comprised 4 μL of 5 × FastPfu buffer, 2 μL of 2.5 mM dNTPs, 10 ng of template DNA, 0.8 μL of each primer, 0.4 μL of FastPfu polymerase, and ddH2O up to 20 μL. PCR conditions included an initial denaturation at 95°C for 3 min, followed by 35 cycles of denaturation at 95°C for 30 s, annealing at 55°C for 20 s, and extension at 72°C for 45 s, with a final extension at 72°C for 10 min. The samples were subsequently maintained at 4°C. Each sample was replicated three times.

#### Illumina MiSeq sequencing

PCR products from the same samples were combined, separated via 2% agarose gel electrophoresis, and purified using the AxyPrep DNA Gel Extraction Kit (Axygen Biosciences, Union City, CA, United States). The purified products underwent further detection by 2% agarose gel electrophoresis and quantification using a Quantus^™^ Fluorometer. Libraries were constructed utilizing the NEXTflex^™^ Rapid DNA-Seq Kit and sequenced on an Illumina MiSeq PE300 platform (Shanghai Meiji Biomedical Technology Co., Ltd.). Sequence data were processed using the QIIME 1.9.1 software package and referenced against the Silva 138 database. OTU clustering was conducted at 97% similarity using USEARCH software, with low-abundance OTU reads (below 20) being filtered out to enhance data quality. Principal coordinate analysis (PCoA), LEfSe, and FAPROTAX functional prediction were conducted on the Majorbio cloud platform.^1^ Raw data were deposited in the NCBI SRA database (Bacteria: PRJNA1123401, Fungi: PRJNA1123453).

#### Processing of illumina MiSeq reads

Raw sequencing data were subjected to quality control using OpenGene’s fastp software (version 0.20.0), followed by sequence assembly using FLASH software (version 1.2.7). The quality control measures included filtering reads with a tail quality below 20, truncating tails using a 50 bp window at an average quality threshold of 20, and removing reads shorter than 50 bp or containing N bases. For assembly, the minimum overlap length was set at 10 bp, with a maximum mismatch ratio of 0.2 in the overlap region. Samples were differentiated based on their barcodes and primers, permitting no mismatches for barcodes and up to two mismatches for primers, while adjusting sequence orientations as necessary. Subsequently, OTU clustering was performed at 97% similarity using UPARSE software (version 7.1), and chimeric sequences were excluded. Finally, each sequence was annotated for species classification using RDP Classifier software (version 2.2) against the Silva 16S rRNA database (version 138), setting the comparison threshold at 70%.

### Statistical analysis of data

At the OTU or phylum taxonomic level with 97% similarity, we utilized mothur^2^ to compute the Chao1 index (which describes microbial community richness), the Shannon diversity index (which describes microbial community diversity), and the relative abundance composition of each microorganism in the samples under varying random sampling (Wang C. et al., 2022; Wang M. et al., 2022; Xu et al., 2022). Curve plots and community bar graphs were generated using R language (version 4.1.1). Differences in microbial community characteristics, such as diversity indices and relative abundances among treatment groups, were analyzed using one-way ANOVA, with the significance threshold set at *p* < 0.05. Pearson correlation analyses to explore potential correlations between inter-root microbial community characteristics and alfalfa growth performance and physiological indicators were conducted using SPSS 26.0 statistical software (IBM SPSS Inc., Chicago, IL, United States), employing a two-tailed test with *p* < 0.05 indicating significant correlation and *p* < 0.01 indicating highly significant correlation.

## Results

### Differences in growth parameters and chlorophyll content among resistant varieties under atrazine stress

**Figure 1:**
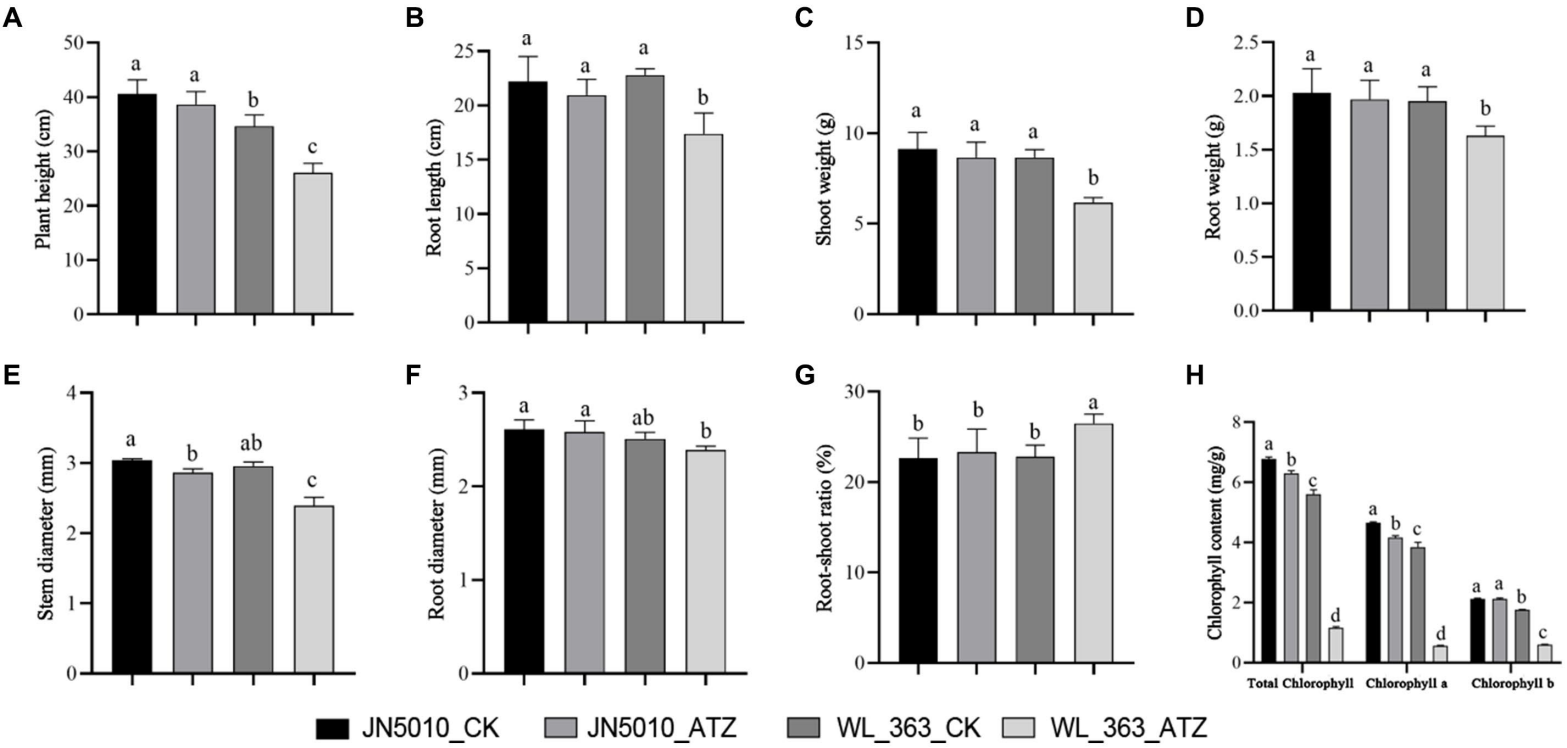
The effects of atrazine stress on morphological changes and photosynthetic parameters in two alfalfa varieties. Plant height **(A)**. Root length **(B)**. Stem and leaf weight **(C)**. Root weight **(D)**. Stem thickness **(E)**. Root thickness **(F)**. Root-shoot ratio **(G)**. Comparison of total chlorophyll, chlorophyll a, and chlorophyll b contents **(H)**. Data represent the mean of three replicates, with error bars indicating standard deviation. Different lowercase letters indicate significant differences between treatments by the Duncan test.

Exposure to atrazine diminished growth parameters in both varieties when compared to the control. Specifically, the variety more tolerant to atrazine, JN5010, exhibited reductions in plant height and root length by 1.96 cm and 1.26 cm, respectively (Figures 1A,B); basal root and stem thickness decreased by 0.03 mm and 0.17 mm, respectively (Figures 1D,E); stem weight and leaf weight were reduced to 0.48 g and 0.06 g, respectively (Figure 1C); and there was a 0.67% increase in the root-to-shoot ratio (Figure 1G). Notably, stem thickness significantly decreased (*p* < 0.05) (Figure 1E), whereas other parameters showed no significant changes. In contrast, the atrazine-sensitive variety, WL-363, was more adversely impacted, with decreases in plant height and root length of 8.58 cm and 5.42 cm, respectively (Figures 1A,B); basal root and stem thickness of 0.12 mm and 0.57 mm, respectively (Figures 1D,E); stem weight and leaf weight of 2.51 g and 0.32 g, respectively (Figure 1C); and a root-to-shoot ratio of 3.68% (Figure 1G). The impact of atrazine stress on chlorophyll content in alfalfa leaves led to a general decrease in chlorophyll levels. In the variety JN5010, both chlorophyll a and total chlorophyll were significantly diminished to 89.48 and 92.77% of control values, respectively (*p* < 0.05), with chlorophyll b levels remaining unaltered. In contrast, variety WL-363 exhibited a significant reduction in chlorophyll content under atrazine stress (*p* < 0.05), with values of chlorophyll a, b, and total chlorophyll reduced to 14.56, 34.66, and 20.86% of the control levels, respectively (Figure 1H).

### Effects of atrazine stress on physiological indicators and enzyme activities of different resistant alfalfa varieties

**Figure 2:**
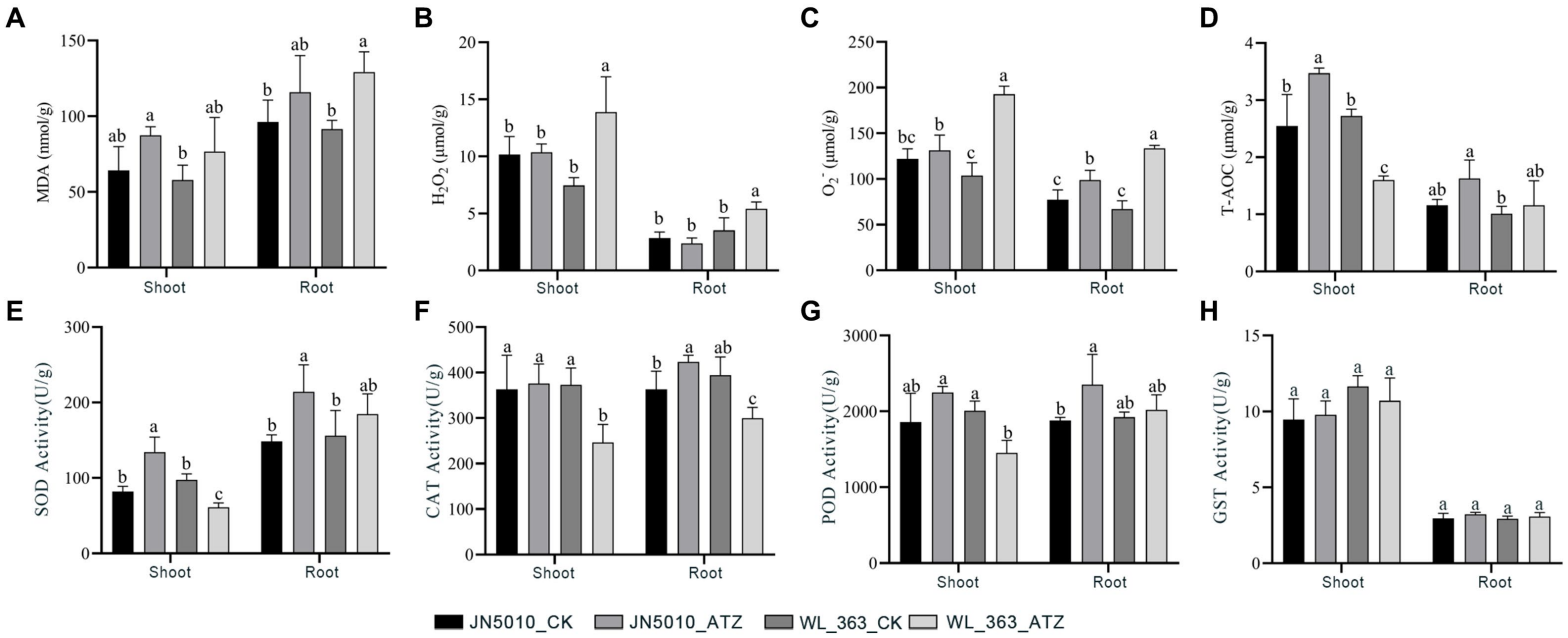
Effects of Atrazine Stress on Oxidative Markers and Enzyme Activities in Shoots and Roots of Two Alfalfa Cultivars. MDA **(A)**, H_2_O_2_ **(B)**, O_2_^−^ **(C)**, T-AOC **(D)**, SOD **(E)**, CAT **(F)**, POD **(G)**, GST **(H)**. Data were means of three replicates. Error bars represented standard deviation. Different letters indicated statistically significant differences at *p* < 0.05.

In the absence of atrazine treatment, there were no significant differences in the levels of malondialdehyde, hydrogen peroxide, and superoxide anion in shoot and root of JN5010 and WL-363. However, under atrazine stress, the malondialdehyde content increased in both varieties. Specifically, in JN5010, the malondialdehyde levels in shoot and roots of the atrazine-treated group were 1.36 and 1.21 times higher than those in the control group, respectively. In contrast, WL-363 exhibited a more pronounced increase under atrazine stress, with malondialdehyde levels in the shoot and roots rising by 1.57 and 1.41 times compared to the control group (Figures 2A–D). In the present study, a slight increase in hydrogen peroxide content was noted in shoot of JN5010 under atrazine stress, whereas a moderate decrease was observed in root. Conversely, the hydrogen peroxide content in both shoot and root of WL-363 exhibited a significant increase (*p* < 0.05) under atrazine stress (Figure 2B). Total antioxidant capacity of alfalfa JN5010 was enhanced in both shoot and root under atrazine stress compared to the control group, with a significant increase (*p* < 0.05) observed in shoot. Conversely, the root changes in WL-363 under atrazine stress mirrored those of JN5010, yet the total antioxidant capacity in shoot significantly decreased under atrazine stress, representing only 58.82% (*p* < 0.05) of the control group (Figure 2D).

Under atrazine stress, the superoxide dismutase (SOD) activity in shoot and root of JN5010 increased to 134.06 and 214.10 U/g, respectively, significantly surpassing the control values of 81.96 and 148.30 U/g (*p* < 0.05). Meanwhile, the SOD activity in WL-363 roots rose by 18.57% under atrazine stress, although this increase was not statistically significant. In contrast, SOD level in shoots significantly decreased to 62.73% of the control value (*p* < 0.05) (Figure 2E). For JN5010, catalase (CAT) activity in root increased significantly to 1.16 times that of the control (*p* < 0.05), with no notable change observed in shoot. Conversely, WL-363 displayed a significant decrease in peroxidase (POD) activity under atrazine stress in both components, falling to 62.73 and 76.03% of control levels (*p* < 0.05), respectively. Notably, the peroxidase activity in JN5010 increased under atrazine stress, with significant alterations observed in root (Figures 2F,G). No significant changes (*p* > 0.05) were observed in the glutathione *S*-transferase (GST) activity of two alfalfa cultivars, JN5010 and WL-363, when subjected to atrazine stress. However, the shoot of JN5010 exhibited a slight increase in GST activity, while a decrease was noted in WL-363 (Figure 2H).

### Atrazine stress effects on Alfalfa’s inter-root bacterial community

**Figure 3:**
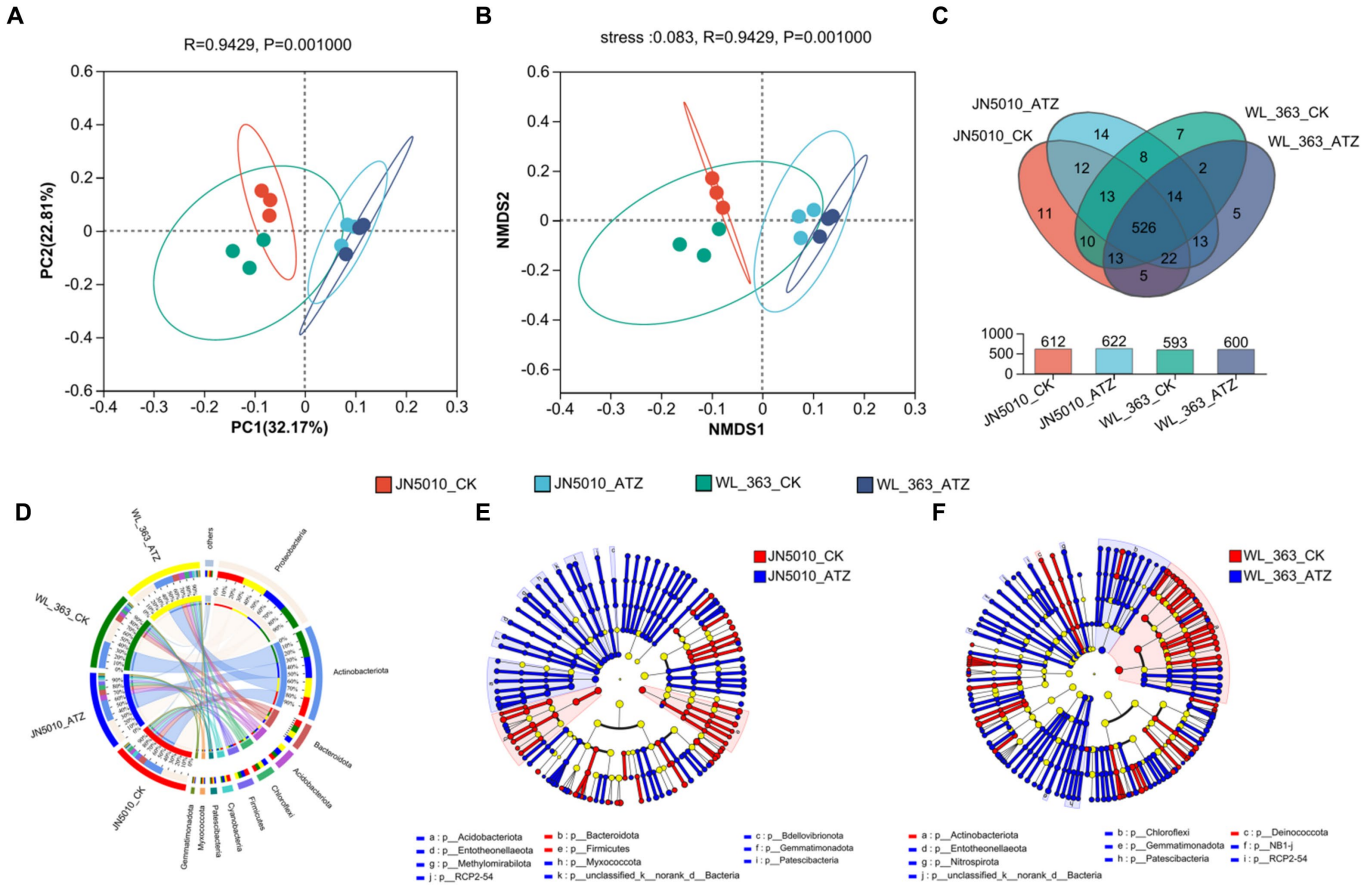
Analysis of rhizosphere bacterial communities under Atrazine stress. **(A)** Principal Coordinate Analysis (PCoA) and **(B)** Non-metric Multidimensional Scaling (NMDS) of core OTUs. **(C)** Venn diagram showing shared and unique core OTUs among treatments. Comparison of bacterial phylum levels in the alfalfa rhizosphere under different treatments **(D)** and the dendrogram of community structure **(E,F)**.

Rank Abundance curves, which arrange microbial sequences by abundance, complemented by dilution curve graphs derived from Alpha diversity indices at varied sequencing depths, are utilized to assess microbial diversity, species richness, and community evenness. A flatter curve indicates a more uniform distribution of species. At 30,000 reads, the curves level off, suggesting a homogeneous species composition across samples and adequate sequencing coverage (Supplementary Figure S2). The Sobs, Ace, and Chao indices, which assess bacterial community abundance, showed significant increases in JN5010 following atrazine application (Supplementary Figures S3A–C). Although WL-363 also exhibited elevated abundance levels, these were not statistically significant (Supplementary Figures S3A–C). The Shannon and Simpson indices, which gage community diversity, indicate that higher Shannon values and lower Simpson values correspond to greater diversity. Under atrazine stress, both JN5010 and WL-363 experienced a significant increase in Shannon diversity and a notable decrease in Simpson diversity for WL-363, highlighting atrazine’s role in enhancing bacterial community diversity within the inter-root zones of alfalfa (Supplementary Figures S3D,E). Principal Component Analysis (PCoA) demonstrates a clear segregation between the inter-root bacterial communities of alfalfa varieties JN5010 and WL-363, with the separation becoming more pronounced upon atrazine treatment (Figure 3A). This suggests that each variety harbors a unique bacterial community composition which responds differently to atrazine stress. The analysis using a Venn diagram (Figure 3C) identified 2,780 bacterial Operational Taxonomic Units (OTUs) across all samples. Specifically, in the JN5010 variety, the control and atrazine-treated groups contained 2,399 and 2,474 OTUs, respectively, while the WL-363 variety contained 2,327 and 2,408 OTUs, respectively, with a shared core of 2020 OTUs across treatments. The analysis revealed distinct bacterial profiles under atrazine stress, with JN5010 displaying 293 unique OTUs in the treated group compared to 218 in the control, and WL-363 exhibiting 316 unique OTUs under treatment versus 235 in the control. These results underscore the PCoA findings of distinct bacterial diversification influenced by atrazine (Figure 3B).

Atrazine treatments exhibited minimal impact on the bacterial composition of inter-root soil in WL-363 and JN5010 alfalfa varieties. The predominant bacterial phyla—*Proteobacteria*, *Actinobacteriota*, *Bacteroidota*, *Acidobacteriota*, *Chloroflexi*, and *Firmicutes*—demonstrated varying abundances across treatments (Figure 3D). In the WL-363 variety, the control group showed abundances of 32.46, 37.37, 8.71, 4.81, 4.65, and 4.44%, respectively, with only minor variations observed in the treated groups. For the JN5010 variety, the control group displayed abundances of 36.98, 26.87, 9.09, 4.71, 4.43, and 6.23%, with treated groups adjusting to 33.02, 29.18, 7.40, 7.53, 6.49, and 4.89% (Supplementary Figure S4). Notably, Chloroflexi, Acidobacteriota, and Firmicutes exhibited similar response patterns to atrazine stress across both varieties, whereas Proteobacteria, Actinobacteriota, and Bacteroidota showed distinct trends. Linear Discriminant Analysis (LDA), as executed by LefSe, assessed the taxonomic compositions of four alfalfa inter-root bacterial community groups to identify those significantly affected by the presence or absence of atrazine stress. The evolutionary dendrograms of these communities (Figures 3D–F) depict yellowish nodes representing microbial taxa with minimal impact on inter-group variances, while red nodes indicate species that are more prevalent in atrazine-free environments. Conversely, blue nodes highlight species that flourish under atrazine stress conditions, with the node size denoting their relative abundance. In the absence of atrazine stress, the inter-root soil of WL-363 was predominantly characterized by the presence of *Actinobacteriota* and *Deinococcus* (Figure 3F), while the soil associated with JN5010 was enriched with *Bacteroidota* and *Firmicutes* (Figure 3E). Upon exposure to atrazine, WL-363’s soil exhibited an increase in *Patescibacteria*, *p_unclassified_k_norank_d-Bacteria*, *Chloroflexi*, *NB1-j*, *Entotheonellaeota*, *Gemmatimonadota*, RCP2-54, and *Nitrospirota* (Figure 3F). In contrast, the inter-root soil of JN5010 showed a significant enrichment of *Myxococcota*, *Methylomirabilota*, *Acidobacteriota*, and *Bdellovibrionota* under similar stress conditions (Figure 3E). Notably, *Chloroflexi* and *Nitrospirota* were significantly present in WL-363’s soil but absent in JN5010’s, highlighting distinct microbial enrichment patterns between the two varieties under atrazine stress.

**Figure 4:**
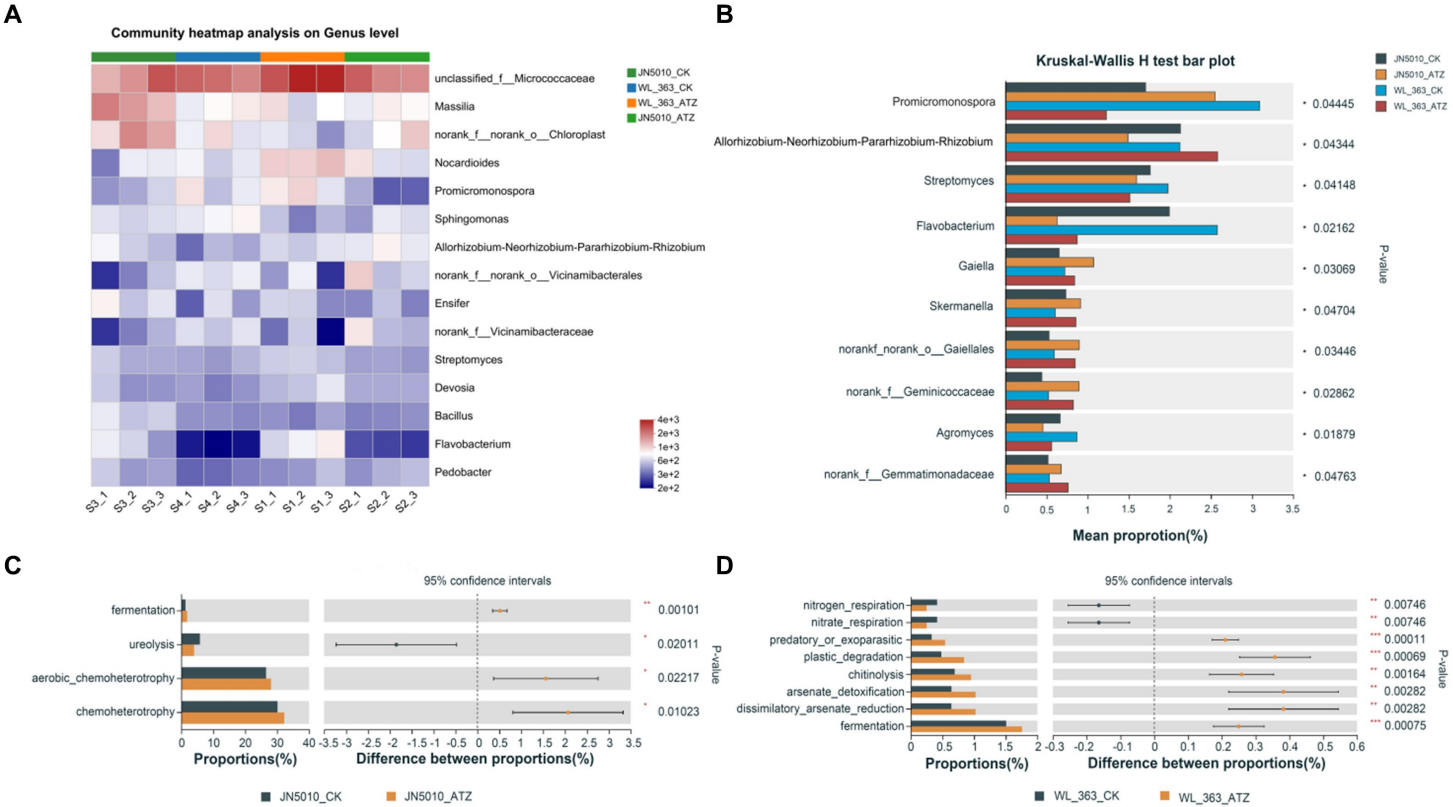
Analysis of differential bacteria and their functions in the rhizosphere of two alfalfa varieties under atrazine stress. **(A)** Genus-level analysis of differential bacteria; S1, S2, S3, and S4 represent WL_363_CK, WL_363_ATZ, JN5010_CK, and JN5010_ATZ treatments, respectively. **(B)** Unique bacterial communities at the genus level in different rhizosphere soil environments under atrazine stress. **(C,D)** Functional analysis of bacterial communities in diverse rhizosphere soils using FAPROTAX (Functional Annotation of Prokaryotic Taxa), highlighting functional diversity and potential ecological roles. WL_363 and JN5010 represent two alfalfa varieties; CK: control group; ATZ: atrazine treatment group.

To investigate the adaptation mechanisms of different alfalfa varieties to atrazine stress, the study analyzed genus-level compositional changes in the soil surrounding the roots of alfalfa varieties JN5010 and WL-363. This analysis revealed significant fine-scale fluctuations in microbial abundance due to atrazine treatment. The dominant genera included *unclassified_f_Micrococcaceae*, *Massilia*, *Nocardioides*, *Promicromonospora*, *Sphingomonas*, *Allorhizobium*, and *Ensifer* (Figure 4A). In the WL-363 control group, the abundances of these genera were 11.71, 2.77, 4.07, 3.10, 1.63, 2.13, and 1.83%, respectively, with slight alterations observed under atrazine treatment. For JN5010, control conditions showed abundances of 6.65, 5.63, 2.02, 1.70, 4.99, 2.19, and 2.13%, which adjusted to 7.48, 2.81, 2.30, 2.55, 2.63, and 1.49% following atrazine exposure (Supplementary Figure S5). *Sphingomonas* and *Ensifer* exhibited consistent response patterns in both varieties, whereas unclassified *Micrococcaceae*, *Nocardioides*, *Massilia*, and *Allorhizobium* displayed varied responses. Specifically, *Sphingomonas* increased, and *Ensifer* decreased under stress; unclassified *Micrococcaceae* and *Nocardioides* increased in JN5010 but decreased in WL-363, highlighting diverse bacterial responses to atrazine across alfalfa varieties. Analysis at the genus level of bacterial community abundances in four inter-root soils of alfalfa revealed species with differential responses. Under atrazine stress, the genera *Streptomyces*, *Flavobacterium*, and *Agromyces* exhibited reduced abundance in both alfalfa varieties, JN5010 and WL-363. In contrast, genera such as *Gemmatimonadaceae*, *Gaiella*, *Skermanella*, *norank_f_norank_o_Gaiellales*, and *norank_f_Geminicoccaceae* experienced an increase in abundance due to atrazine exposure. Notably, *Promicromonospora* showed a decrease in WL-363 but an increase in JN5010. Conversely, the *Allorhizobium-Neorhizobium-Pararhizobium-Rhizobium* complex demonstrated an inverse response pattern, highlighting distinct interspecies variations in response to atrazine stress (Figure 4B). Utilizing the FAPROTAX database, which links prokaryotic taxa with specific metabolic or ecologically significant functions (such as nitrification and denitrification), we analyzed functional disparities in bacterial communities between alfalfa varieties JN5010 and WL-363 under atrazine stress (Figures 4C,D). WL-363 exhibited a broader range of affected functions, including predatory or ectoparasitic activities, plastic degradation, chitinolysis, arsenate detoxification, dissimilatory arsenate reduction, and fermentation (Figure 4D). Notably, WL-363 demonstrated a decrease in nitrate respiration functions, in contrast to JN5010, which displayed up-regulation of these functions in response to atrazine. Conversely, JN5010 showed enhanced functions related to fermentation, aerobic chemoheterotrophy, and chemoheterotrophy, along with a decrease in ureolysis, highlighting distinct functional adaptations to atrazine stress across the two varieties (Figure 4C).

### Changes in the inter-root fungal community of alfalfa under atrazine stress

**Figure 5:**
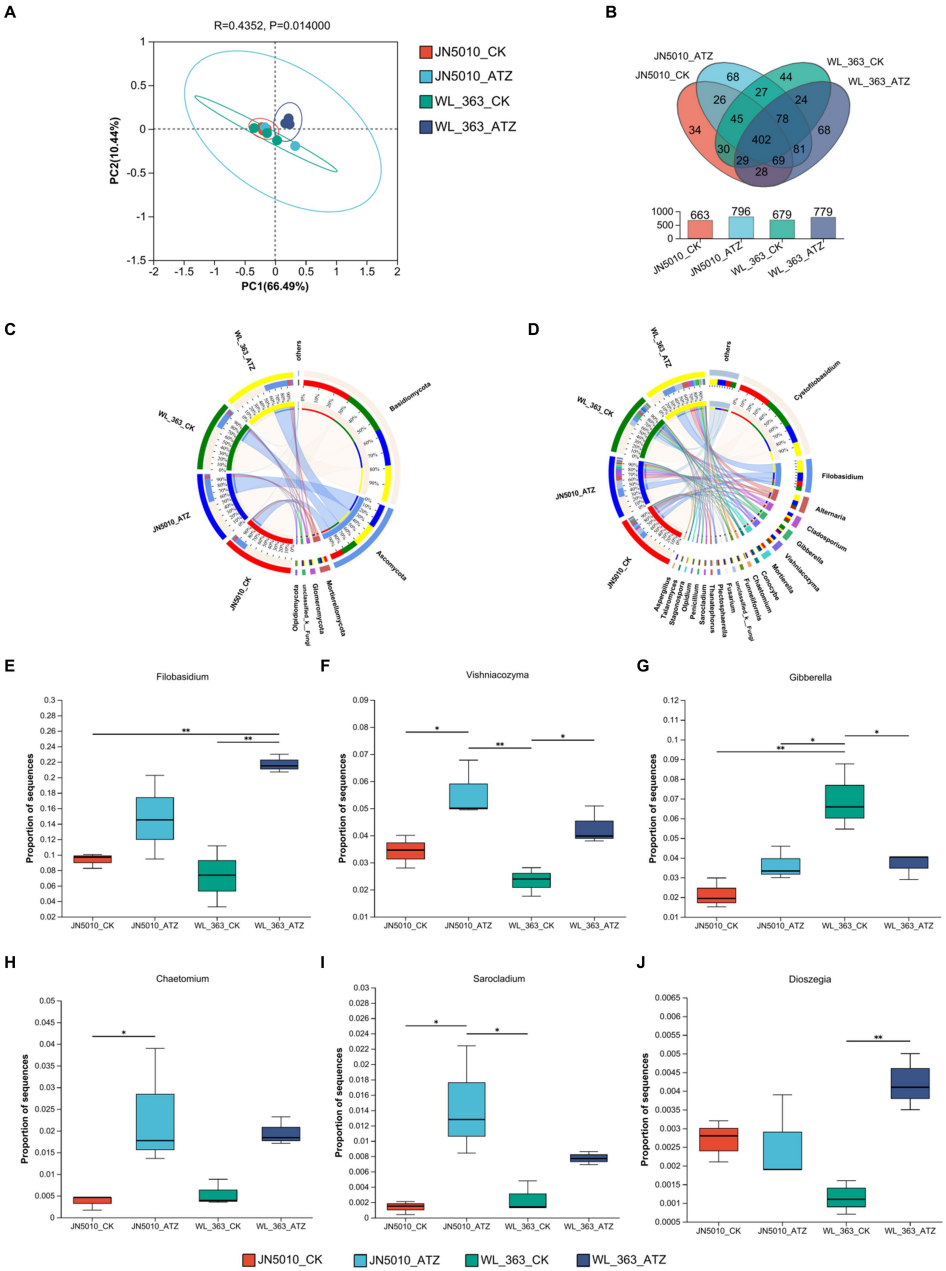
Analysis of rhizosphere fungal communities under atrazine stress. **(A)** Principal Coordinate Analysis (PCoA) of core OTUs of rhizosphere fungi. **(B)** Venn diagram showing shared and unique core OTUs among treatments. **(C)** Comparison of fungal community composition at the genus level in the alfalfa rhizosphere under different treatments. **(D)** Detailed analysis of fungal genera abundance across treatments. **(E–J)** Analysis of differential abundance of fungal genera between treatments under atrazine stress. Differences marked with * and ** are significant at *p* < 0.05 and 0.01, respectively. OTU, Operational Taxonomic Unit.

When the sample reaches 45,000 reads, the curve flattens, indicating homogeneity in species composition and adequate sequencing depth (Supplementary Figure S6). Supplementary Figures S7A–C show that atrazine treatment slightly increased the Sobs, Chao, and Ace indices for WL-363’s inter-root fungi. Concurrently, JN5010 exhibited an increase in richness as evidenced by enhanced Sobs and Chao indices. The Shannon and Simpson indices, which measure community diversity, suggest that higher Shannon values and lower Simpson values correspond to greater diversity. Under atrazine stress, both JN5010 and WL-363 displayed higher Shannon indices and lower Simpson indices, indicating increased diversity in their inter-root fungal communities (Supplementary Figures S7D,E). However, the improvements in fungal richness and diversity did not achieve statistical significance (*p* > 0.05). PCoA analysis revealed greater overlap in fungal communities than in bacterial communities across inter-root soils, with slight group-to-group species variation (Figure 5A). Venn diagram analysis was conducted to explore the core fungal Operational Taxonomic Units (OTUs) within the inter-root soils of alfalfa varieties JN5010 and WL-363. This analysis identified a total of 1,053 fungal OTUs. For JN5010, the control and atrazine-treated groups contained 663 and 796 OTUs, respectively. In contrast, WL-363 had 679 and 779 OTUs in its control and treated groups, respectively. The shared OTUs between the WL-363 control and treated groups amounted to 533. The unique OTU counts for the WL-363 groups were 246 and 146, respectively, indicating a distinct fungal community shift under atrazine stress. Similarly, JN5010 exhibited 542 total OTUs across treatments, with unique OTU counts of 254 and 121 for the control and treated soils, respectively. These findings underscore the extent to which atrazine stress alters the composition of fungal species in the inter-root zones of these alfalfa varieties (Figure 5B).

In four different inter-root soils, the predominant fungal taxa were *Basidiomycota*, *Ascomycota*, *Mortierellomycota*, *Glomeromycota*, *unclassified_k_Fungi*, and *Olpidiomycota* (Figure 5C). For JN5010, the control group abundances were 74.47, 18.94, 4.23, 0.61, 0.91, and 0.43%, respectively; these values shifted in the treated group to 57.02, 36.28, 2.32, 1.87, 0.91, and 0.09%. In WL-363, control group abundances were 67.50, 25.16, 2.72, 1.55, 1.29, and 1.42%, changing in the treatment group to 55.38, 35.49, 3.76, 2.82, 1.36, and 0.62% (Supplementary Figure S8). Atrazine stress significantly increased the prevalence of *Ascomycota*, *Glomeromycota*, and *Olpidiomycota*, while decreasing that of *Basidiomycota* in both alfalfa varieties. Notably, *Mortierellomycota* abundance decreased in JN5010 but increased in WL-363 under atrazine stress, indicating differential responses of the fungal communities between the varieties (Figure 5C). The fungal genus composition in the rhizosphere soils of alfalfa cultivars JN5010 and WL-363 exhibited significantly different responses to atrazine stress. Dominant genera included *Cystofilobasidium*, *Filobasidium*, *Alternaria*, *Cladosporium*, *Gibberella*, *Vishniacozyma*, *Mortierella*, *Conocybe*, *Chaetomium*, and *Funneliformis* (Figure 5D). Following atrazine treatment, the changes in fungal abundance in JN5010 were 31.49, 14.74, 8.06, 6.21%, among others, whereas in WL-363, they were 24.26, 21.73, 10.89, 4.68%, respectively (Supplementary Figure S9). Atrazine stress resulted in a reduced prevalence of *Cystofilobasidium*, while it increased that of *Filobasidium*, *Alternaria*, *Cladosporium*, and others in both cultivars. Interestingly, *Gibberella* and *Conocybe* showed an increase in abundance in JN5010’s soil but a decline in WL-363, reflecting an inverse trend for *Mortierella* between the cultivars, thus highlighting atrazine’s specific effects on fungal community dynamics (Figure 5D). Differences in fungal genera abundance were observed at the genus level, based on comparisons between the inter-root fungal populations of two species under both treatment and control conditions. Under atrazine stress, WL-363 showed significant increases in the abundance of *Filobasidium*, *Vishniacozyma*, and *Dioszegia* (*p* < 0.05), and a significant decrease in *Gibberella* (*p* < 0.05), compared to the control. Conversely, the inter-root soil of JN5010 exposed to atrazine demonstrated increased levels of *Filobasidium*, *Vishniacozyma*, *Gibberella*, *Chaetomium*, and *Sarocladium*, with the increases in *Vishniacozyma*, *Chaetomium*, and *Sarocladium* being statistically significant (*p* < 0.05) (Figures 5E–J).

### Correlation analysis of JN5010/WL-363 inter-root microorganisms with alfalfa growth and physiological indexes

**Figure 6:**
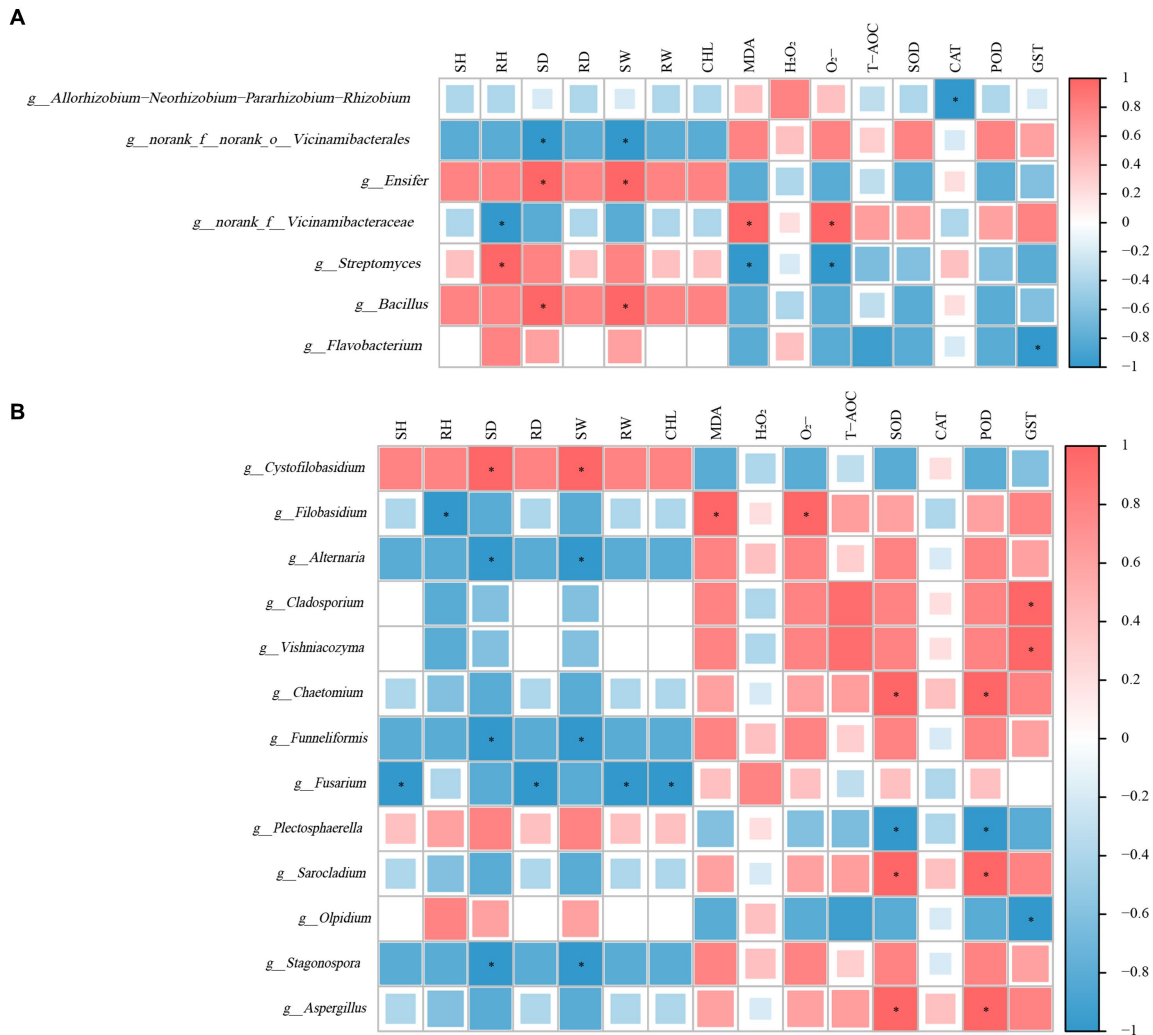
Correlation analysis of microbial abundance with alfalfa growth and stress tolerance indices under atrazine stress. **(A)** Correlation of bacterial genera showing differential abundance between treatments with alfalfa growth parameters and stress tolerance indicators. **(B)** Correlation of fungal genera abundance with alfalfa growth performance and physiological indices. Correlations were calculated using Pearson’s correlation coefficient. Statistical significance is indicated at *p* < 0.05 (*).

The correlation analysis between alfalfa growth, stress tolerance indices, and the abundance of specific bacterial genera revealed significant relationships. Genera *g_Ensifer* and *g_Bacillus* were positively correlated with the development of alfalfa roots, as well as stem and leaf growth, exhibiting particularly strong correlations with stem thickness and biomass (*p* < 0.05). Conversely, malondialdehyde content, superoxide anion content, and activities of SOD and POD were negatively correlated with these growth parameters. The abundance of genus *g_Streptomyces* correlated positively with root length but was inversely related to oxidative stress markers such as malondialdehyde and superoxide anion content (*p* < 0.05). Similarly, the abundance of *g_norank_f_Vicinamibacteraceae* was negatively associated with plant growth but displayed significant positive correlations with malondialdehyde and superoxide anion contents and stress adversity indicators (*p* < 0.05) (Figure 6A).

Correlation analyses between inter-root fungi, growth performance, and physiological indices of alfalfa revealed that the fungal genera *g_Altemaria, g_Funneliformis*, and *g_Stagonospora* were significantly negatively correlated (*p* < 0.05) with stem thickness and above-ground biomass. In contrast, *g_Aspergillus*, *g_Sarocladium*, and *g_Chaetomium* exhibited significant positive correlations (*p* < 0.05) with root superoxide dismutase and peroxidase activities. Excluding *g_Cystofilobasidium*, *g_Olpidium*, and *g_Plectosphaerella*, the majority of abundant fungal genera displayed negative correlations with vegetative growth indices, while showing positive correlations with oxidative damage indices and enzyme activities (Figure 6B).

## Discussion

### Growth indices of different resistant varieties under atrazine stress

Phenotypic variations serve as primary indicators of plant responses to pesticide stress, elucidating tolerance levels. Studies have demonstrated that atrazine disrupts weed proliferation by inhibiting photosynthesis, specifically by blocking electron transport in photosystem II (PS II), which results in leaf discoloration and necrosis, ultimately stunting plant growth (Hess and Hess, 2000; Fan et al., 2018). Chlorophyll, essential for photosynthesis, often decreases under pesticide stress, evidenced by leaf yellowing (Fan et al., 2018; Castro et al., 2022; Kai et al., 2023). The effect of atrazine on chlorophyll levels in various crops has been extensively documented; for example, Yu et al. (2021) noted that the total chlorophyll content in maize leaves declined with increasing atrazine concentration. A study on algae found that atrazine treatment inhibited algal chlorophyll production (Hu et al., 2021) and observed a reduction in chlorophyll and carotenoid levels in zucchini seedlings subjected to higher atrazine concentrations. Similar effects, alongside diminished plant growth, have been confirmed in alfalfa (Zhang et al., 2017; Fan et al., 2018; Yang et al., 2022). Our study indicated that atrazine exposure significantly decreased chlorophyll content in the sensitive alfalfa variety WL-363, with reductions in chlorophyll a, b, and total chlorophyll to 14.56, 34.66, and 20.86% of the control, respectively. This exposure also significantly reduced plant height by 24.75%, stem thickness by 9.56%, and stem and leaf weight by 28.98%, consistent with prior findings on atrazine’s effects on alfalfa. Conversely, the highly resistant JN5010 variety showed no significant change in plant height or stem and leaf weight under atrazine stress, except for a notable decrease in stem thickness by 5.59%. These findings highlight the variance in tolerance to atrazine among alfalfa varieties, with resistant types maintaining average growth and development under such conditions.

In direct contact with the soil, the root system serves as the primary pathway for the entry of pesticides, particularly soil pesticide residues, into plants (Song et al., 2023). Atrazine, a systemic herbicide that inhibits photosynthesis, exhibits variable effects on root development. Alberto et al. (2022) found that atrazine treatment significantly inhibited the emergence of roots in *Arabidopsis thaliana*. Yu et al. (2021) observed a decreasing trend in the fresh weight of maize seedling roots as the concentration of atrazine increased. Jia et al. (2021) noted that atrazine influenced the development of sweet potato roots, although the effect was not significant compared to controls. In our study, the root growth of the atrazine-resistant JN5010 did not significantly differ from the control. However, in the sensitive WL-363, root length and weight were substantially reduced to 76.23 and 83.59% of the control, respectively, highlighting the specific impact of atrazine on the root growth of sensitive varieties. These findings are consistent with those of Yang et al. (2022) and Zhang et al. (2016). Although contrasting with Zhang et al. (2016), our results indicate that the root system was comparatively less affected by atrazine, with more pronounced effects observed in the aboveground parts of alfalfa under stress.

In order to further explore the molecular mechanism of alfalfa’s resistance to atrazine stress, based on transcriptomics, we identified some potential differentially expressed genes (DEGs) related to atrazine degradation and detoxification, such as ABC transporters, cytochrome 450, and glutathione *S*-transferase (Supplementary Table S1, unpublished). Furthermore, ABC transporters, essential transmembrane proteins, are involved in cellular metabolism and toxin efflux, playing a crucial role in plant responses to pesticide stress, particularly herbicides, by facilitating various detoxification processes (Yuan et al., 2007; Conte and Lloyd, 2011; Hwang et al., 2016; Busi et al., 2018). Studies indicate that upregulation of specific ABC transporters correlates with increased herbicide tolerance in *Amaranthus palmeri* (Peng et al., 2010). In this study, we identified 24 DEGs encoding ABC transporters were prominently upregulated under atrazine stress (Supplementary Figure S10A), which were supposed to be related to the transmembrane transport of atrazine. Additionally, cytochrome P450 enzymes, a critical class of multifunctional oxidoreductases, play key roles in plant metabolism, detoxification, and response to environmental stresses (Pandian et al., 2020). Specific P450 subtypes in alfalfa effectively metabolize atrazine, converting it into less toxic metabolites through biochemical processes such as oxidation and dealkylation, thus significantly reducing its adverse effects on plants (Zhang et al., 2014, 2016). In the current study, 20 cytochrome P450s genes were identified and significantly upregulated under atrazine stress (Supplementary Figure S10B), indicating that these genes might be involved in the detoxification of atrazine. Moreover, Glutathione *S*-transferases (GSTs) are crucial for plant to response to environmental stimuli and detoxification (Anik et al., 2024). Plants typically increase the gene expression and activity of GSTs under adverse conditions, enhancing their antioxidant capability and resistance to toxins (Chronopoulou et al., 2017). Previous studies have demonstrated that homo-glutathione (hGSH) levels could conjugate atrazine in alfalfa (Zhang et al., 2014). Similarly, 11 GSTs-coding genes were identified and upregulated under atrazine exposure in this study (Supplementary Figure S10C), which were considered to be associated with the potential for degradation of atrazine.

### Degree of oxidative damage under atrazine stress in different resistant varieties

Beyond its influence on photosynthesis and carbon fixation, atrazine’s ecotoxicity significantly increases reactive oxygen species (ROS) within plants, leading to oxidative stress that may culminate in lipid peroxidation, cell membrane damage, and potential plant death. Extensive research has shown that these toxic effects are not confined to target organisms; they also induce oxidative damage in non-target plants and soil microorganisms (Ramel et al., 2009; Zhang Y. et al., 2012; Jiang et al., 2016; Fan et al., 2018; De Andrade et al., 2019). The measurement of malondialdehyde content is a standard metric for assessing lipid peroxidation and quantifying oxidative damage. The excessive accumulation of ROS is the primary catalyst for oxidative stress and lipid peroxidation (Jiang et al., 2016; Farooq et al., 2019), with numerous studies exploring ROS’s role in plant stress responses and tolerance (Sierla et al., 2013; Das and Roychoudhury, 2014; Choudhury et al., 2017). Prior research has documented an increase in reactive oxygen species, such as hydrogen peroxide, in plants subjected to external stressors, accompanied by increased malondialdehyde content. For instance, Mahrookashani et al. (2017) found significant increases in malondialdehyde levels in plants experiencing drought stress. Similarly, Zhang J. J. et al. (2012) noted a noteworthy rise in malondialdehyde content in rice plants subjected to atrazine treatment. In summary, extensive research indicates frequent coincidences of plant stress injury with accumulating reactive oxygen species (ROS). This study revealed that atrazine stress significantly increased superoxide anion and hydrogen peroxide levels in the sensitive variety WL-363. Concurrently, malondialdehyde (MDA) levels in both leaves and roots of WL-363 increased under atrazine stress to 1.57 and 1.41 times that of the control, respectively. This suggests that atrazine stress led to an excessive buildup of reactive oxygen species in the stems, leaves, and roots of the sensitive WL-363 alfalfa variety, resulting in oxidative damage. Zhang et al. (2017) reported that after 6 days of atrazine exposure at 0.08 mg/L, the MDA levels in the aboveground parts and roots surged to 1.58 and 2.46 times the control, respectively, indicating severe oxidation of membrane lipids. This finding aligns with our experiment’s results. Conversely, in the atrazine-stressed JN5010, malondialdehyde levels were 1.36 and 1.21 times higher than the control in aboveground parts and roots, respectively, without significant changes in hydrogen peroxide and superoxide anion levels. This suggests that the highly tolerant JN5010 cultivar experienced minor oxidative damage but maintained average growth under atrazine stress.

### Alfalfa responds to atrazine stress through changes in enzyme activity

Alfalfa’s response to atrazine stress is mediated through enzymatic activity alterations, with antioxidant enzymes playing a pivotal role in scavenging excess reactive oxygen species (ROS) to maintain intracellular homeostasis (Jin et al., 2020). Superoxide dismutase (SOD), a crucial antioxidant enzyme, mitigates damage from free radicals and ROS induced by various stresses (Stephenie et al., 2020), converting superoxide radicals into less harmful hydrogen peroxide. Subsequently, enzymes such as peroxidase (POD) and catalase (CAT) facilitate the conversion of hydrogen peroxide into water and molecular oxygen (Wu et al., 2017). This experiment demonstrated that atrazine stress significantly enhanced SOD, CAT, and POD activities in the roots of JN5010. Meanwhile, WL-363 displayed a moderate increase in SOD and POD activities and a notable decrease in CAT activity.

Moreover, the aboveground parts of JN5010 exhibited a significant increase in antioxidant enzyme activities under stress, in contrast to WL-363, where the activities of all three enzymes were markedly reduced. These findings indicate that JN5010 effectively mitigates oxidative damage through enhanced peroxidase activities, particularly in aboveground tissues, while WL-363 shows a decrease in aboveground antioxidant defense, suggesting that the oxidative stress exceeds its antioxidant capacity. This activation and subsequent depletion of antioxidant mechanisms under stress reflect findings from numerous studies (Li et al., 2012; Jiang et al., 2016), demonstrating a dynamic where initial antioxidant responses are overwhelmed by oxidative damage once reactive oxygen species (ROS) accumulation exceeds the plant’s resistance threshold.

### Microbial communities of different resistant varieties under atrazine stress

Soil microorganisms play a pivotal role in soil ecology, profoundly influencing the growth of terrestrial plants through complex interactions (Galloway et al., 2008; Berendsen et al., 2012; Li et al., 2021). These organisms enhance soil fertility by facilitating nutrient transport to the vicinity of plant roots, mediated by plant-induced biochemical processes (White et al., 2018). Moreover, plants regulate the soil microbial community by producing sugars, amino acids, and reactive oxygen species, underscoring a dynamic regulatory relationship (Ge et al., 2023). This interaction primarily occurs in the inter-root zone, which is a critical area mediating the intricate ecological network between soil, microbes, and plants. It plays an essential role in nutrient supply, improving plant stress tolerance, and facilitating pollutant degradation within the plant–soil-microbial system (Bonkowski et al., 2009; Mendes et al., 2013). Pesticide residues modify soil conditions, thereby altering the microbial community composition in the inter-root zone and indirectly affecting plant health through changes in the root system (Sun et al., 2016; Huang et al., 2021). This study observed an increased diversity and abundance of bacteria and fungi in the inter-root zones of two alfalfa varieties treated with atrazine compared to controls, consistent with previous findings. Beta diversity analysis showed less variation among fungal communities across four inter-root soils in PCoA plots, while bacterial groups displayed more significant differences. Specifically, inter-root bacterial communities of both varieties under atrazine-free conditions demonstrated some separation, which became more pronounced with atrazine exposure, indicating that atrazine stress alters the bacterial composition in a variety-specific manner.

The microbial community structure in the inter-root soils of four samples showed no significant differences in the dominant bacterial and fungal phyla. The prevalent bacterial phyla included *Proteobacteria*, *Actinobacteriota*, *Bacteroidota*, *Acidobacteriota*, *Chloroflexi*, and *Firmicutes*, while the principal fungal groups were *Basidiomycota*, *Ascomycota*, *Mortierellomycota*, *Glomeromycota*, and *Olpidiomycota*. Under atrazine stress, specific phyla such as *Actinobacteriota*, *Myxococcota*, *Methylomirabilota*, and *Nitrospirabilota* exhibited notable variations in abundance between the two alfalfa varieties. The Nitrospirabilis phylum, in particular, increased in WL-363 but decreased in JN5010, indicating an alteration in nitrogen cycling within the inter-root environment of WL-363 due to modified soil nitrogen utilization. In contrast, the *Actinobacteria* phylum was significantly up-regulated in JN5010, reflecting its resilience and beneficial role in plant-microbe interactions, potentially contributing to its robust atrazine resistance. Notably, *Actinobacteria*, known for enhancing plant growth and mitigating abiotic stress, have demonstrated potential in stress response modulation (Alekhya and Gopalakrishnan, 2017; Bhatti et al., 2017; Soumare et al., 2019). For instance, treatment with the *Actinobacteria* strain Act12 was reported to decrease oxidative stress markers and enhance defense hormone levels and resistance gene expression in tomatoes (Li et al., 2019). These findings highlight the complex interplay between soil microorganisms and plant resilience mechanisms under atrazine stress.

Echoing gate-level analysis findings, atrazine stress did not significantly alter the composition of dominant fungal and bacterial genera in the inter-root soils across four samples. The abundance of *Ensifer* was notably reduced in the inter-root soils of both alfalfa varieties due to atrazine exposure. While extensive research recognizes *Spermatomyces* spp. for their atrazine-degrading capabilities (Chen et al., 2017; Ma et al., 2017), this study observed a distinctive pattern: the prevalence of *Spermophilus* spp. was consistently higher in JN5010 compared to WL-363, irrespective of atrazine treatment. Additionally, the presence of *Serratia* spp. varied between the two alfalfa varieties under stress, with a decrease in JN5010 and an increase in WL-363. *Serratia marcescens* spp., commonly found in soils impacted by heavy metals and prolonged fertilizer use (Zhang et al., 2006; Zheng et al., 2017), have been recognized for their potential to degrade pollutants such as polycyclic aromatic hydrocarbon phenanthrene and amide herbicides (Lou et al., 2016; Wang et al., 2016). However, their ability to break down atrazine residues remains undetermined.

In our study, the abundance of *Streptomyces* in the inter-root soil of WL-363 was notably lower under atrazine stress compared to JN5010, highlighting the significant role of *Streptomyces* spp. in plant growth enhancement within the *Actinobacteria* phylum and its ability to increase atrazine degradation rates (Olanrewaju and Babalola, 2019; Saez et al., 2022). Similarly, *Agromyces*, belonging to the same *Actinomycetes* phylum as *Streptomyces*, showed comparable trends in abundance under atrazine stress. In contrast, research on *Gaiella* spp. has primarily focused on the removal of heavy metals, *dimethylamine*, and *dibenzofuran,* with no studies on triazine herbicide residues (Raj et al., 2019; Almnehlawi et al., 2021; Mathivanan et al., 2021). However, this experiment recorded a significant increase in *Gaiella* abundance in response to atrazine stress, consistent with findings that temperature stress may also enhance *Gaiella* abundance (Miao et al., 2021), and its persistence in mercury-contaminated soils over extensive periods (Li et al., 2022). This implies a general responsiveness of *Gaiella* to external stressors by increasing its abundance, whereas *Rhizobium* spp. abundance decreased in JN5010 but increased in WL-363 under atrazine stress. Previous research has shown that *Rhizobium* spp. inoculation improves growth parameters, photosynthetic pigments, stomatal morphology, and antioxidant enzyme activities in soybeans grown in fly ash-amended soils (Hussain and Faizan, 2023). The elevated presence of *Rhizobium* spp. in atrazine-stressed WL-363, compared to the control, suggests a potential atrazine-induced oxidative damage in WL-363, activating plant-microbe defense mechanisms which were less likely invoked in JN5010 due to reduced oxidative stress.

Different fungal abundance at the genus level was observed in the inter-root fungi of two alfalfa varieties under control and atrazine stress conditions. Specifically, genera such as *Filobasidium*, *Vishniacozyma*, and *Dioszegia* demonstrated increased abundance under atrazine exposure, predominantly from the phylum *Stamenomycetes*. Notably, *Dioszegia*, known for reducing bacterial colonization efficiency in *Arabidopsis thaliana*, serves as a central node in microbial networks, facilitating *host-microbiome* interactions (Agler et al., 2016; Trivedi et al., 2020). Similarly, the genus Stretch yeast has been identified as a mediator for microbial community interactions (McCormack et al., 1994). Conversely, the abundance of *Erythrobacter* spp. WL-363 in the inter-root soil decreased under atrazine stress, while that of JN5010 increased. Despite its known pathogenic properties, *Erythrobacter* spp. secrete *gibberellins*, which are crucial for plant development and stress tolerance (Keswani et al., 2022; Ptošková et al., 2022; Tian et al., 2022). This experiment aligns with previous findings that the core microbial flora remains stable under specific environmental or plant influences (Garrido-Oter et al., 2018), yet environmental factors can still significantly affect microbial community structure (Du et al., 2018; Hamonts et al., 2018; Muller et al., 2018). Although atrazine stress did not alter the high abundance composition of bacterial groups, it did affect the abundance ratios within the *Actinomycetes* and *Stramenomycetes* phyla, impacting the microbial community structure. Correlation analysis revealed positive associations between fungal genera *g_Ensifer*, *g_Bacillus*, and *g_Streptomyces* with plant growth, and negative correlations with oxidative stress markers (Zhang et al., 2006; Zheng et al., 2017). Conversely, *g_norank_f_Vicinamibacteraceae* showed a negative correlation with plant growth but a positive association with stress indicators, suggesting its abundance increases with atrazine-induced alfalfa damage. Most fungal genera were positively correlated with plant oxidative damage, indicating that the toxic effect of atrazine on alfalfa plants, in terms of inhibiting plant growth performance, is responded to by many fungal genera.

## Conclusion

This study investigated the adaptive responses of atrazine-resistant alfalfa cultivars, specifically comparing the resistant cultivar JN 5010 with the sensitive cultivar WL-363 in terms of physiological responses and root-associated microbial community dynamics under atrazine stress. The research further revealed that WL-363 experiences significant oxidative stress, whereas JN5010 exhibits enhanced antioxidant enzyme activity, effectively neutralizing reactive oxygen species levels and sustaining normal alfalfa growth. Despite atrazine exposure, the composition of bacterial and fungal communities in the rhizosphere of these cultivars remained stable. However, a noticeable shift in the abundance of microbes associated with atrazine degradation and resistance was observed, suggesting distinct microbial adaptation strategies between the two cultivars.

## Data availability statement

The raw data supporting the conclusions of this study will be made available by the authors without undue reservation. The data presented in this research have been deposited in the NCBI Sequence Read Archive (SRA) database, with the following accession numbers: Bacteria (PRJNA1123401) and Fungi (PRJNA1123453).

## Author contributions

YL: Conceptualization, Data curation, Formal analysis, Investigation, Methodology, Writing – original draft, Writing – review & editing. JL: Conceptualization, Data curation, Formal analysis, Investigation, Methodology, Writing – review & editing. CD: Methodology, Project administration, Writing – review & editing. HW: Methodology, Project administration, Writing – review & editing. BL: Data curation, Writing – review & editing. DL: Data curation, Writing – review & editing. YC: Resources, Writing – review & editing. ZW: Resources, Writing – review & editing. SM: Resources, Writing – review & editing. YS: Resources, Writing – review & editing. CW: Data curation, Writing – review & editing. XZ: Resources, Writing – review & editing. HS: Funding acquisition, Writing – review & editing.

## Footnotes

^1^http://www.majorbio.com

^2^http://www.mothur.org/wiki/Calculators
