# Supplementary material for: Physiological and biochemical characteristics and microbial responses of Medicago sativa (Fabales: Fabaceae) varieties with different resistance to atrazine stress
Source: Front Microbiol. 2024 Aug 16;15:1447348. doi: 10.3389/fmicb.2024.1447348 (PMC11363823; doi:10.3389/fmicb.2024.1447348)
Supplement: Supplementary file 1 [file Table_1.DOCX]

# Supplementary Figures


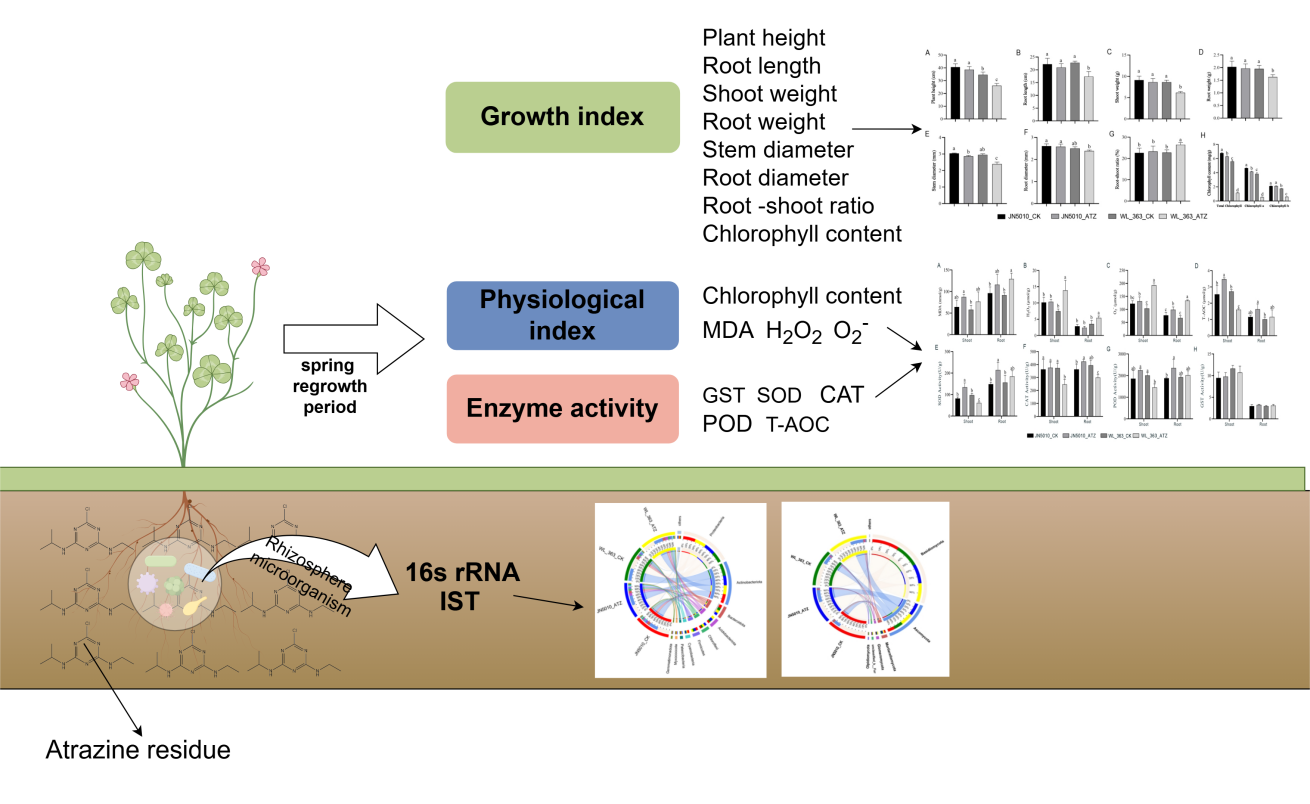


**Supplementary Figure 1** Schematic representation of the experimental approach to investigate atrazine tolerance mechanisms in alfalfa varieties.


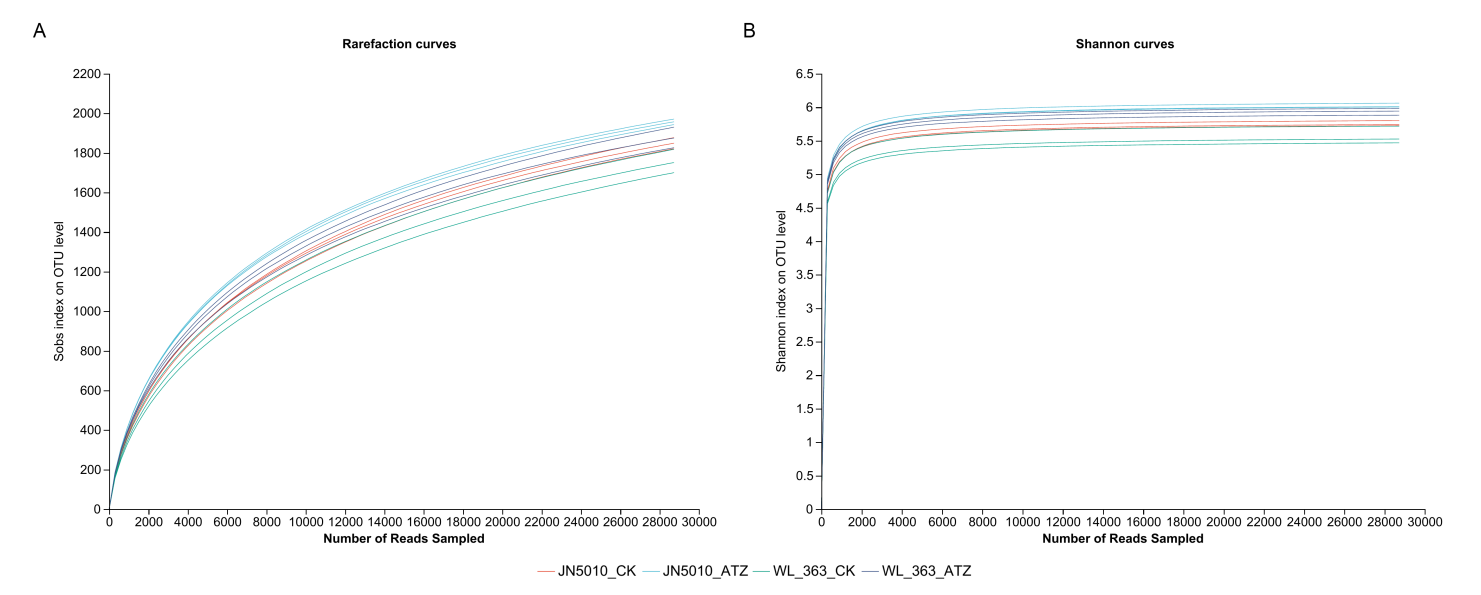


**Supplementary Figure 2** Evaluation of rhizosphere bacterial community diversity in alfalfa under atrazine stress. **(A)** Species richness assessed using the Sobs (observed species) index. **(B)** Community diversity evaluated using the Shannon index. JN5010 and WL_363 represent two different alfalfa varieties. CK: control group; ATZ: atrazine treatment group. This analysis aims to reveal the impact of atrazine on bacterial community diversity in the alfalfa rhizosphere.


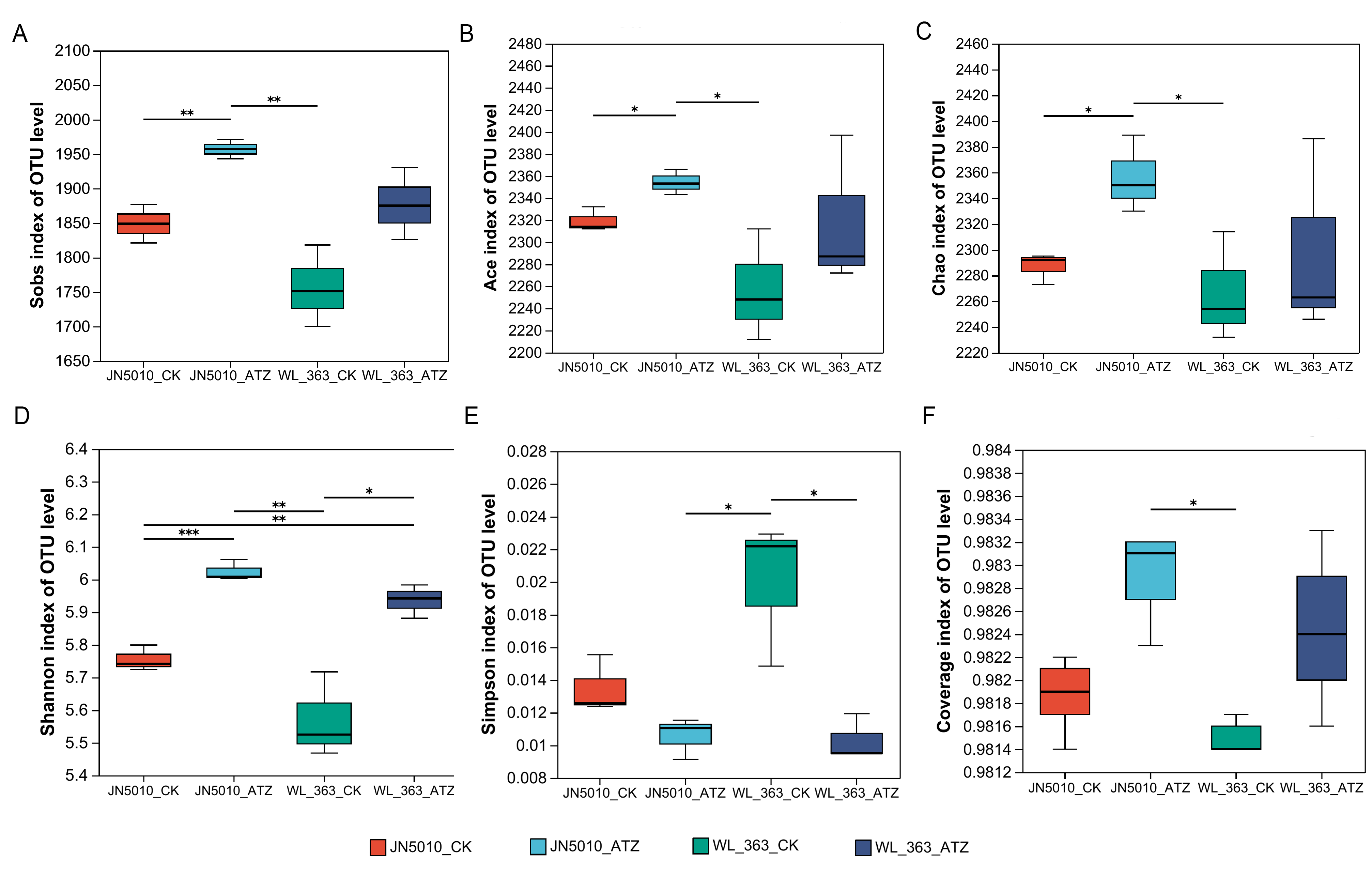


**Supplementary Figure 3** Changes in the bacterial community of alfalfa rhizosphere under atrazine stress. Species richness was estimated using the Sobs **(A)**, Ace **(B)**, and Chao **(C)** indices. Bacterial community diversity was determined using the Shannon **(D)** and Simpson **(E)** indices. Sequencing coverage was assessed using the Coverage index **(F)**. Differences marked with * and ** are significant at *P* < 0.05 and 0.01, respectively.


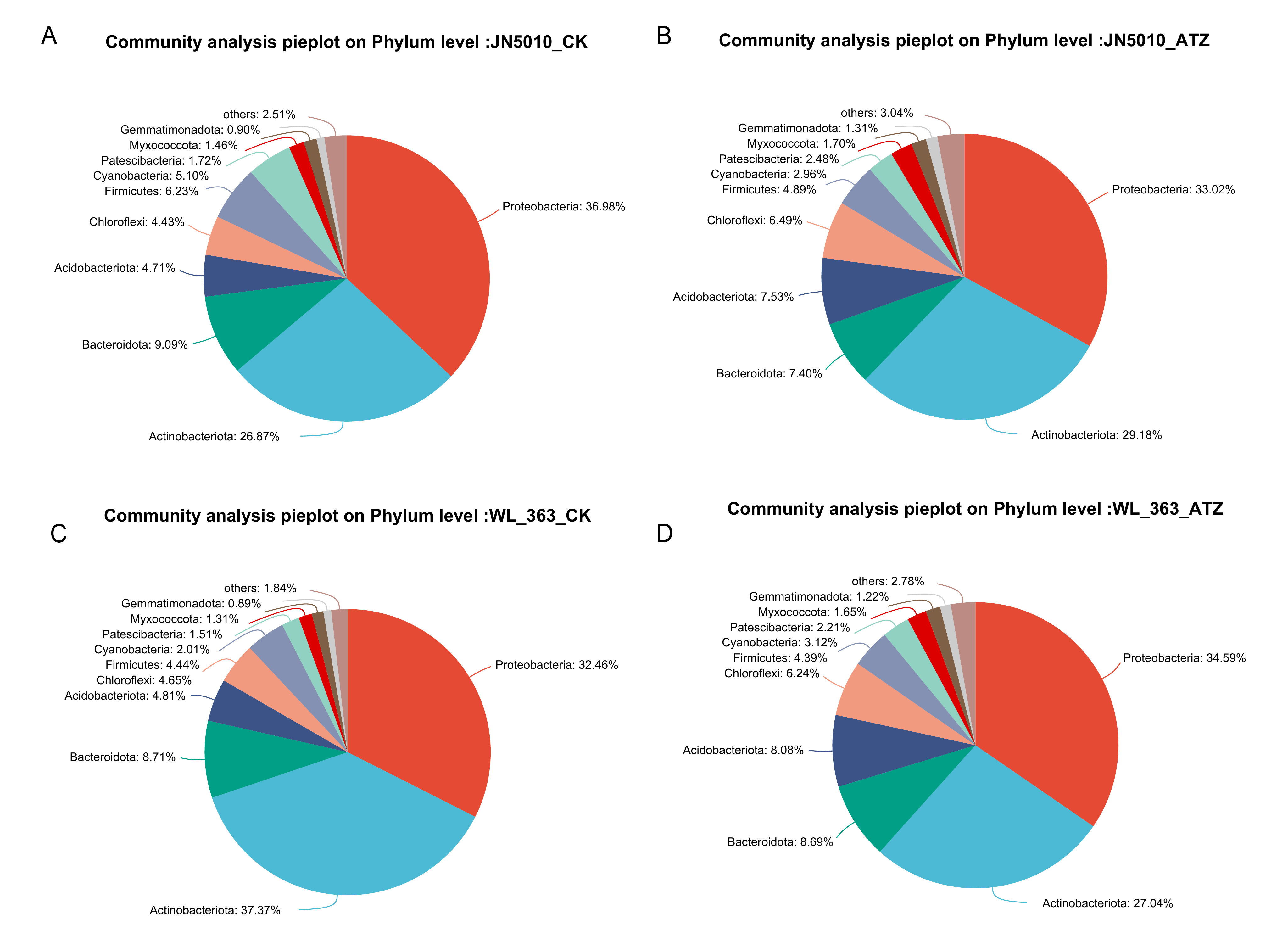


**Supplementary Figure 4** Comparative analysis of abundance levels of dominant bacteria at the phylum level in the rhizosphere of two alfalfa varieties under atrazine stress. **(A)** JN5010_CK (Control), **(B)** JN5010_ATZ (Atrazine treatment), **(C)** WL_363_CK (Control), **(D)** WL_363_ATZ (Atrazine treatment). JN5010 and WL_363 represent two different alfalfa varieties. CK: control group; ATZ: atrazine treatment group. This analysis aims to reveal the impact of atrazine on the composition of dominant bacterial phyla in the alfalfa rhizosphere.


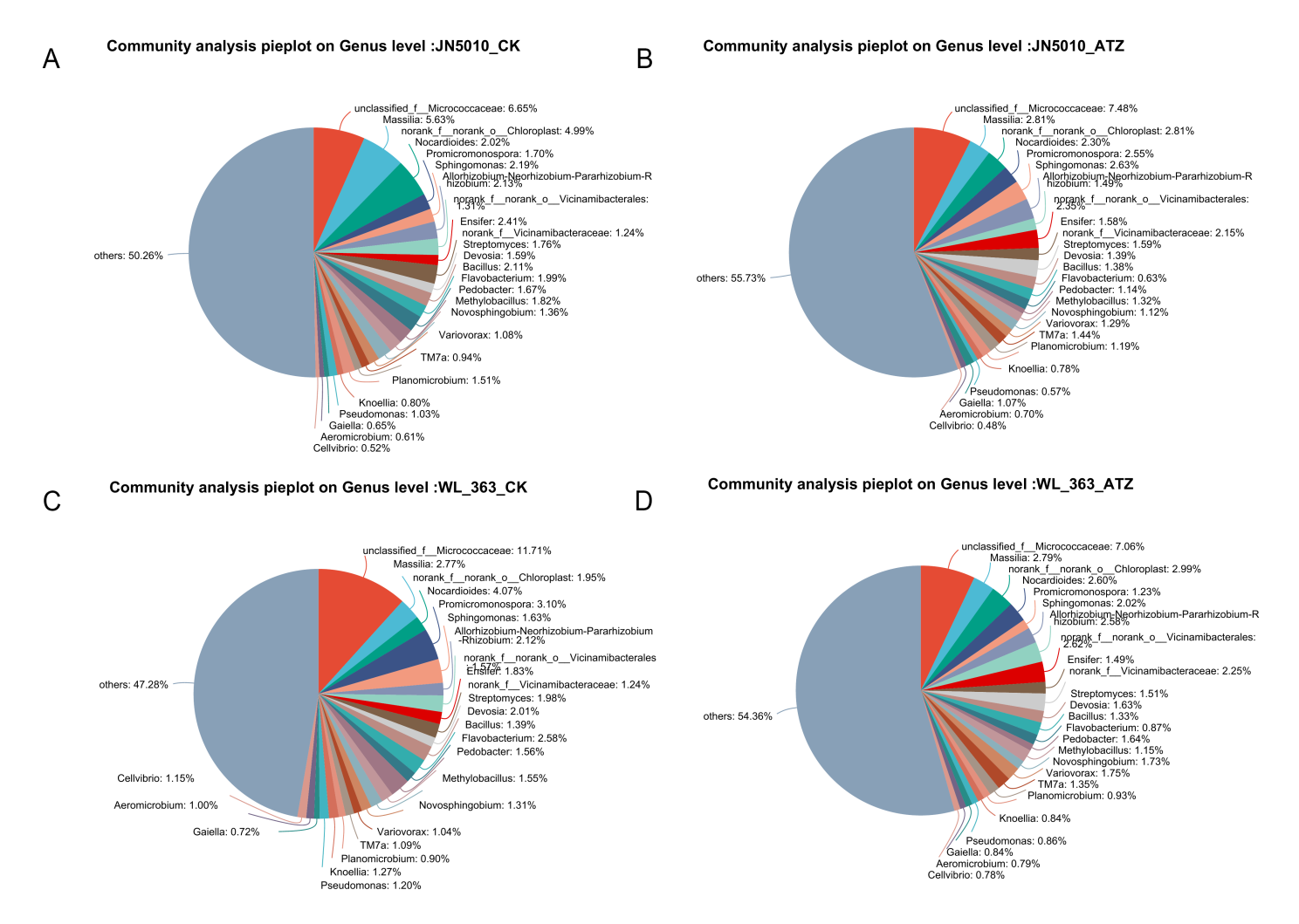


**Supplementary Figure 5** Comparative analysis of abundance levels of dominant bacteria at the genus level in the rhizosphere of two alfalfa varieties under atrazine stress. **(A)** JN5010_CK (Control), **(B)** JN5010_ATZ (Atrazine treatment), **(C)** WL_363_CK (Control), **(D)** WL_363_ATZ (Atrazine treatment). JN5010 and WL_363 represent two different alfalfa varieties. CK: control group; ATZ: atrazine treatment group. This analysis aims to reveal the impact of atrazine on the composition of dominant bacterial genera in the alfalfa rhizosphere.


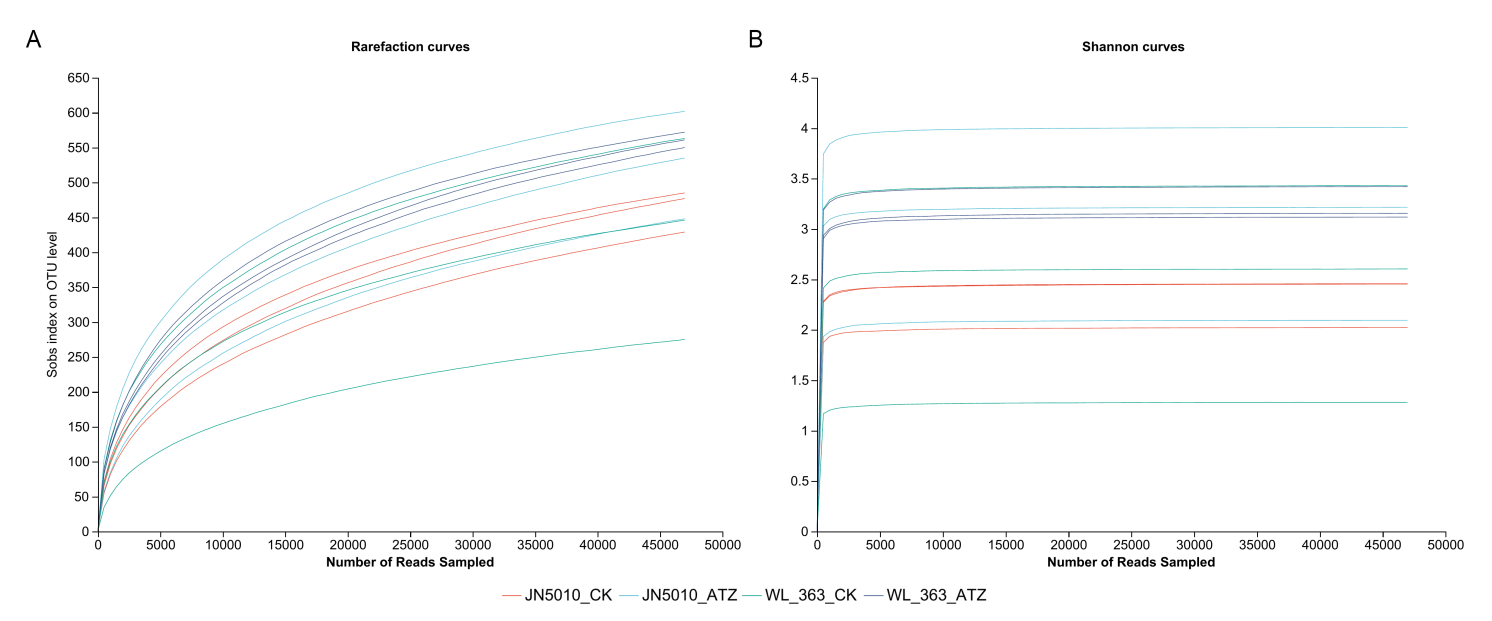


**Supplementary Figure 6** Evaluation of rhizosphere fungal community diversity in alfalfa under atrazine stress. **(A)** Species richness assessed using the Sobs (observed species) index. **(B)** Community diversity evaluated using the Shannon index. JN5010 and WL_363 represent two different alfalfa varieties. CK: control group; ATZ: atrazine treatment group. This analysis aims to reveal the impact of atrazine on fungal community diversity in the alfalfa rhizosphere.


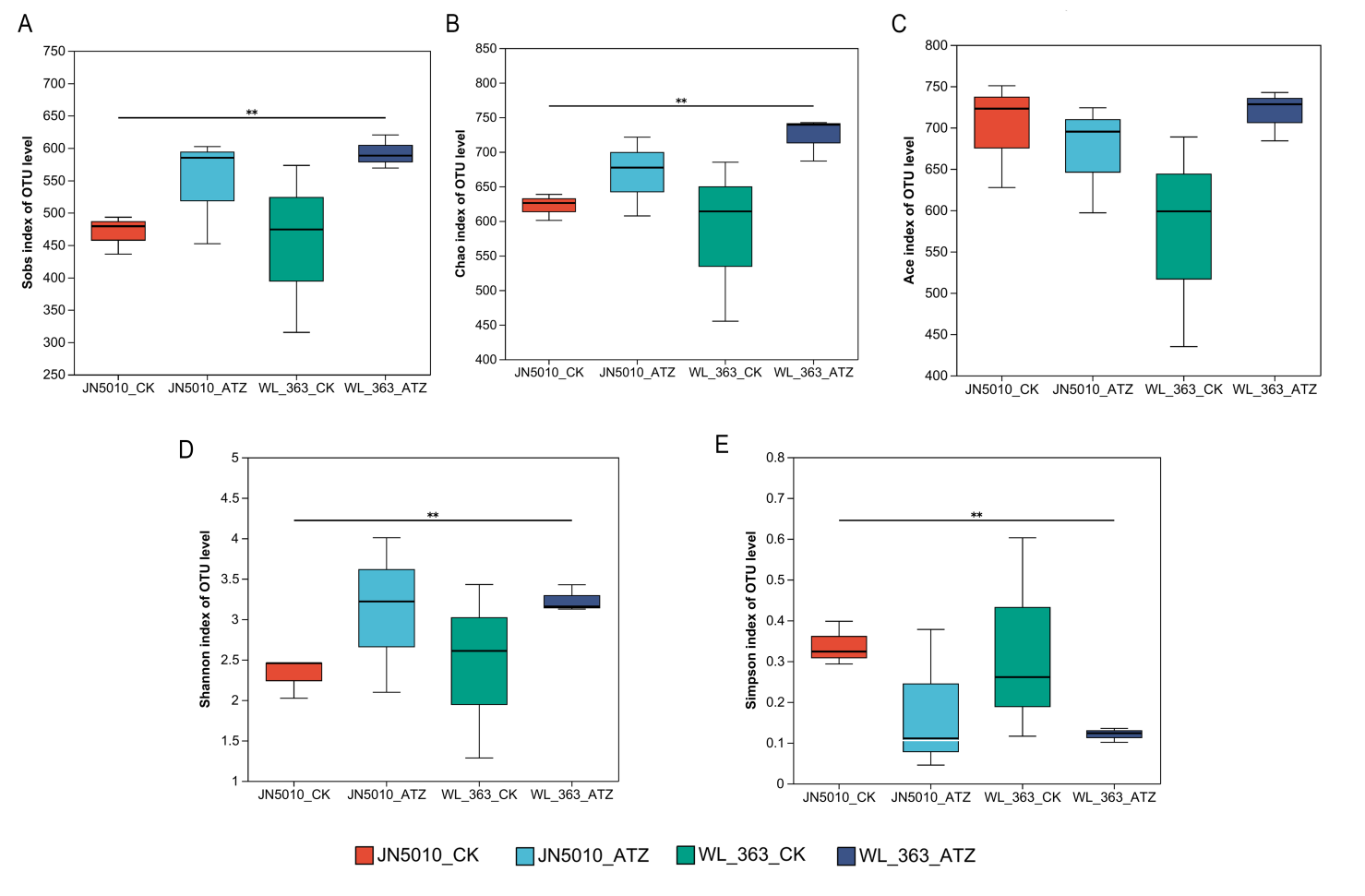


**Supplementary Figure 7** Changes in fungal communities in the rhizosphere of alfalfa under atrazine stress. Fungal species richness was quantified using Sobs index **(A)**, Chao index **(B)**, and Ace index **(C)**. Fungal community diversity was evaluated using Shannon index **(D)** and Simpson index **(E)**. JN5010 and WL_363 represent two different alfalfa varieties. CK: control group; ATZ: atrazine treatment group. This analysis aims to reveal the impact of atrazine on fungal community structure and diversity in the alfalfa rhizosphere.


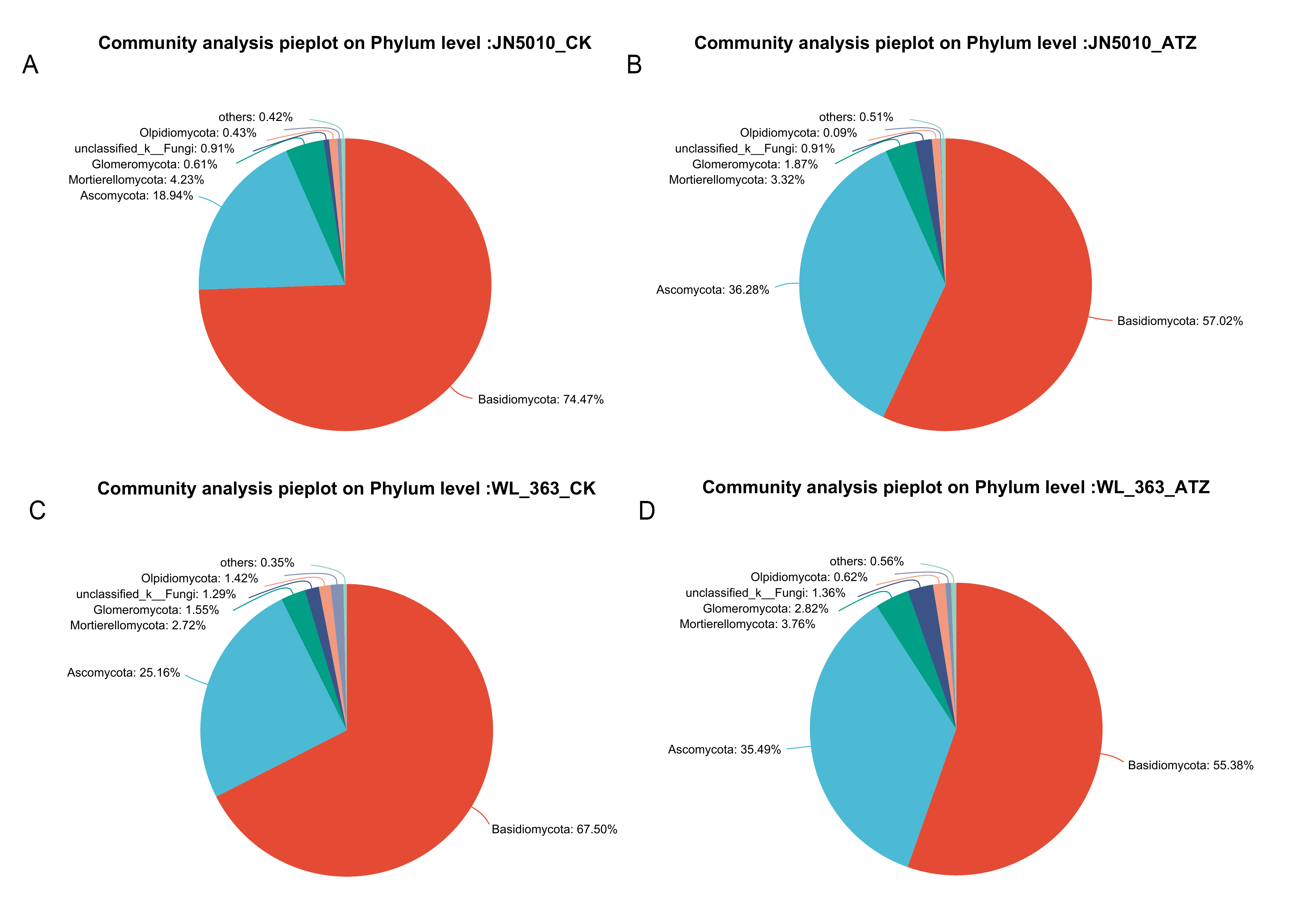


**Supplementary Figure 8** Comparative analysis of the abundance levels of dominant fungi at the phylum level in the rhizosphere of two alfalfa varieties under atrazine stress. **(A)** JN5010_CK (Control), **(B)** JN5010_ATZ (Atrazine treatment), **(C)** WL_363_CK (Control), **(D)** WL_363_ATZ (Atrazine treatment). JN5010 and WL_363 represent two different alfalfa varieties. CK: control group; ATZ: atrazine treatment group. This analysis aims to reveal the impact of atrazine on the composition of dominant fungal phyla in the alfalfa rhizosphere.


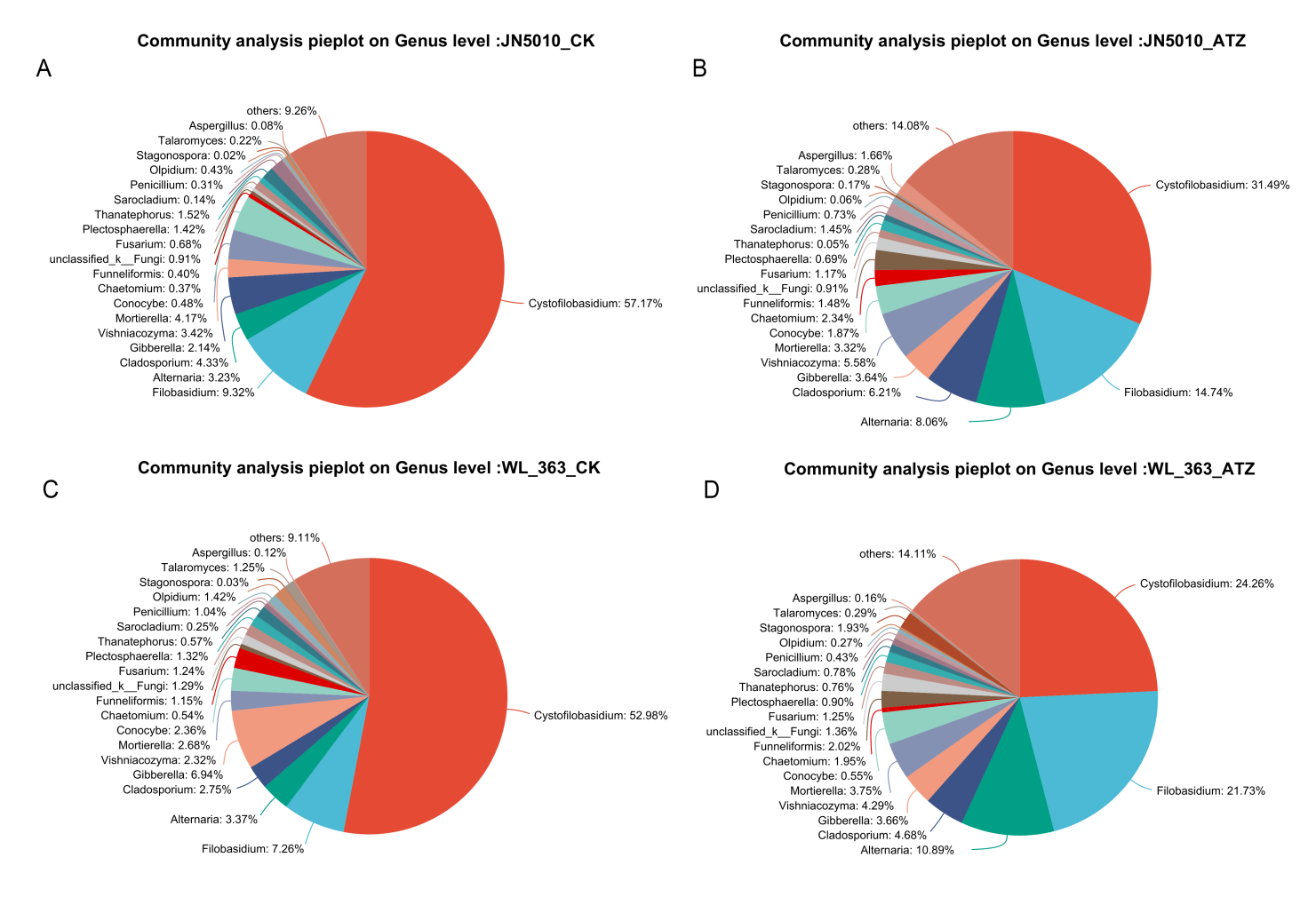


**Supplementary Figure 9** Comparative analysis of the abundance levels of dominant fungi at the genus level in the rhizosphere of two alfalfa varieties under atrazine stress. **(A)** JN5010_CK (Control), **(B)** JN5010_ATZ (Atrazine treatment), **(C)** WL_363_CK (Control), **(D)** WL_363_ATZ (Atrazine treatment). JN5010 and WL_363 represent two different alfalfa varieties. CK: control group; ATZ: atrazine treatment group. This analysis aims to reveal the impact of atrazine on the composition of dominant fungal genera in the alfalfa rhizosphere.


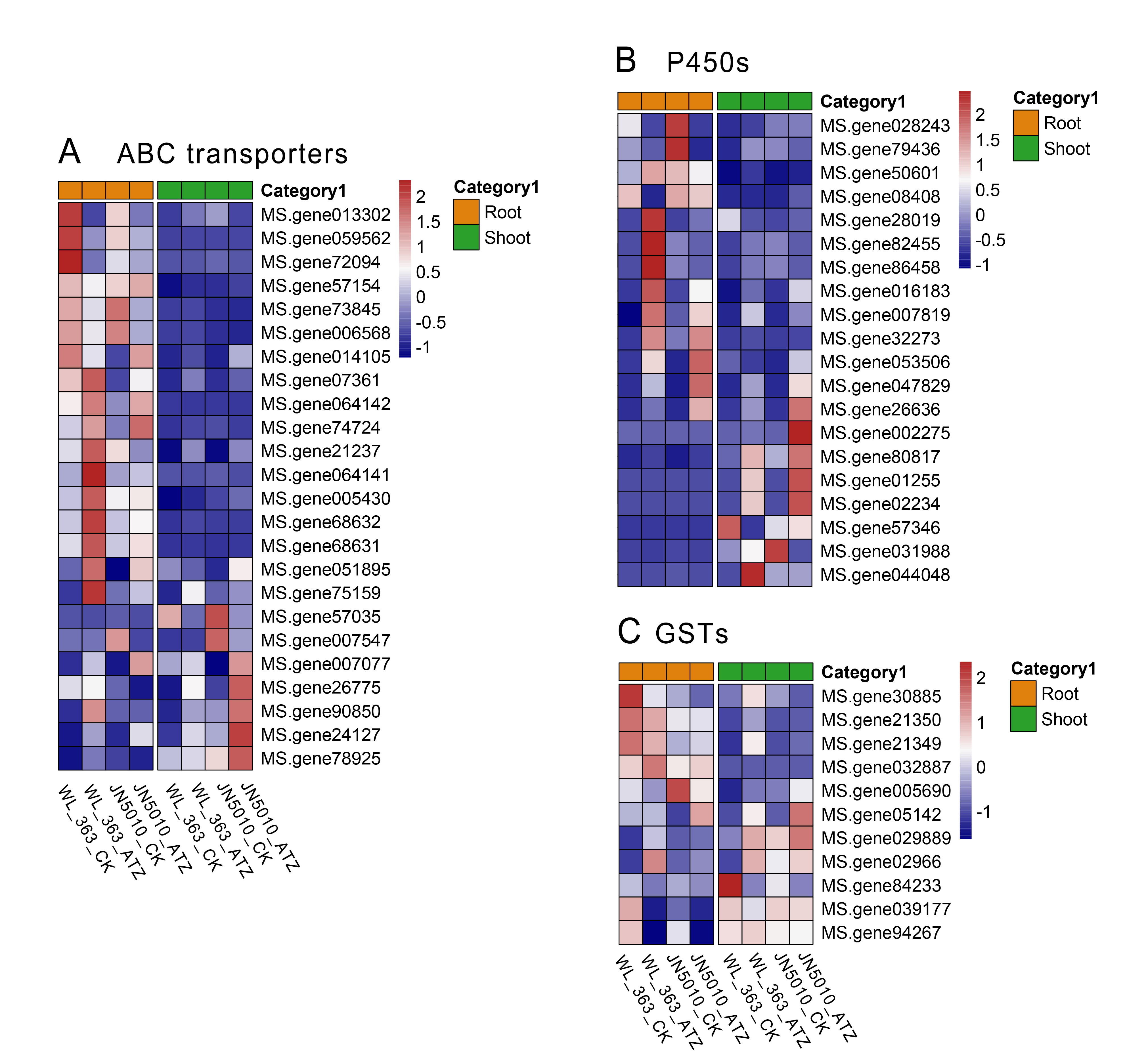


**Supplementary Figure 10** The heatmap of differentially expressed genes (DEGs) possibly related to atrazine detoxification identified in shoots and roots. The gene normalized signal intensities are shown using a log_10_ scale. The red color represents a high gene expression level, while the blue color represents a low gene expression level. (A) ATP-binding cassette transporters (ABC transporters); (B) cytochrome P450s (CYP450s); (C) Glutathione *S*-transferases (GSTs). The transcripts ID of each DEGs is shown on the right.
